# Triazine-Based Graphitic Carbon Nitride Thin Film as a Homogeneous Interphase for Lithium Storage

**DOI:** 10.1021/acsnano.3c08771

**Published:** 2024-01-09

**Authors:** Zihan Song, Jing Hou, Emeline Raguin, Angus Pedersen, Enis Oǧuzhan Eren, Evgeny Senokos, Nadezda V. Tarakina, Paolo Giusto, Markus Antonietti

**Affiliations:** † Colloid Chemistry Department, Max Planck Institute of Colloids and Interfaces, Potsdam 14476, Germany; ‡ Biomaterials Department, Max Planck Institute of Colloids and Interfaces, Potsdam 14476, Germany; § Department of Chemical Engineering, Imperial College London, SW7 2AZ London, U.K.; ∥ Department of Materials, Imperial College London, SW7 2AZ London, U.K.

**Keywords:** Two-dimensional material, Graphite carbon nitride, Triazine-based structure, Thermal vapor deposition, Thin film, Lithium storage

## Abstract

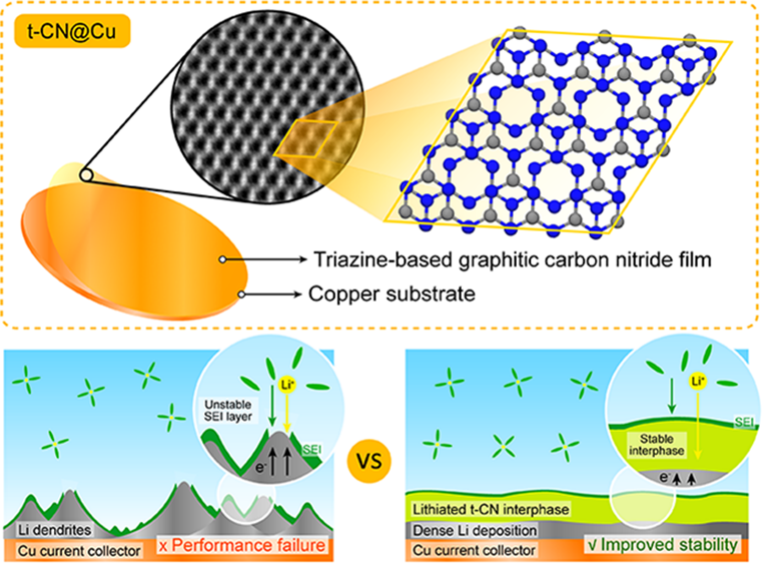

Triazine-based graphitic carbon nitride is a semiconductor material constituted of cross-linked triazine units, which differs from widely reported heptazine-based carbon nitrides. Its triazine-based structure gives rise to significantly different physical chemical properties from the latter. However, it is still a great challenge to experimentally synthesize this material. Here, we propose a synthesis strategy via vapor-metal interfacial condensation on a planar copper substrate to realize homogeneous growth of triazine-based graphitic carbon nitride films over large surfaces. The triazine-based motifs are clearly shown in transmission electron microscopy with high in-plane crystallinity. An AB-stacking arrangement of the layers is orientationlly parallel to the substrate surface. Eventually, the as-prepared films show dense electrochemical lithium deposition attributed to homogeneous charge transport within this thin film interphase, making it a promising solution for energy storage.

## Introduction

Two-dimensional (2D) materials have attracted broad research interest due to their distinctive structural characteristics and physical, chemical, and mechanical properties. The discovery of graphene has promoted the extending exploration of other monoelemental (e.g., boronphene, silicene, etc.) and bi- or multielemental 2D materials (e.g., transition metal sulfides and oxides, hexagonal boron nitride, MXenes, etc.).^1−6^ Among those, carbon nitrides (CNs) are constituted by layers of heterocyclic C–N units cross-linked by nitrogen bonds stacked in a graphitic fashion, with an ideal carbon-to-nitrogen ratio of 0.75.^7,8^ Since their rediscovery in the early 2000s, CNs have been thoroughly investigated for a wide range of applications particularly in energy conversion, such as metal-free photocatalysts and (photo)electrocatalysts and more.^9−11^ However, in most cases, these applications involve the use of CNs as bulk materials, making them impractical for use as optical materials, as membranes for selective separation, and in interfacial modification for battery electrodes, requiring the development of homogeneous thin film coatings over large surfaces.^12−15^ Furthermore, several different structures of CNs have been reported experimentally both based on heptazine units (i.e., tri-s-triazine, C_6_N_7_), such as melon, poly(heptazine imide)s, heptazine-based graphitic carbon nitride, and triazine (C_3_N_3_) units, such as poly(triazine imide)s and triazine-based graphitic carbon nitride.^16^ The latter in particular has recently gained interest, as it provides a structure with significantly smaller repeating units, implying smaller in-plane trigonal voids. Several theoretical studies have shown that the graphitic triazine-based CN (t-CN) has a lower thermodynamic stability with respect to the graphitic heptazine-based CN by 23 kJ/mol.^17,18^ Recently, a method for the synthesis of t-CN has been reported by Algara-Siller et al. by calcination of dicyandiamide in LiBr:KBr (52:48 wt %) in a closed ampule system, leading to poly(triazine imide)/Li^+^Br^–^ as the major phase suspended in the eutectic salt.^19^ The formation of t-CN occurred at the gas-solid and solid-liquid interface as an orange to dark-brown film depending on the reaction conditions.^19^ Electron microscopy characterization and theoretical modeling confirmed that the structure is composed—potentially but not exclusively—of graphitic t-CN stacked in an ABC fashion (interlayer distance 3.29 Å), with the triazine units superimposed to the bridging nitrogen and followed by a void. However, the lowest energy structure among graphitic t-CN was found to be the AB stacking, with a smaller energy difference with respect to the AA and ABC arrangements (14 meV/atom) and with a minimum interlayer distance of 3.22 Å. Electrical conductivity measurements of t-CN flakes revealed a preferential out-of-plane electrical conductivity with respect to the in-plane one, making it of interest for use in electronic device fabrication.^20^

Heptazine-based CN films have been also explored for membrane separation and modification of electrode-electrolyte interphases due to their high ionic conductivity while being essentially impermeable for most organic solvents.^13,21−24^ For these applications, the control over the size of the structural voids and the homogeneity of charge transport are of primary importance, as these affect the ionic transport across the thin film. However, t-CN was never explored in these directions, and a synthetic method to prepare homogeneous, large area, and crack-free t-CN thin is still lacking.

Herein, we propose a strategy for synthesizing the t-CN thin film directly on a planar copper foil as the substrate, denoted as t-CN@Cu. This involves a vapor condensation at the vapor-metal interface in a simple semiclosed system. This thermal vapor deposition proceeded at a relatively low condensation temperature of 500 °C for 4 h to form a homogeneous and crystalline t-CN film in centimeter scale. We deem the Cu substrate to play a fundamental role in the growth and stabilization of the t-CN structure. Indeed, the 2D covalent network grows parallel to the Cu surface with an AB stacking along the *c*-axis. Furthermore, the Cu substrate can be quantitatively etched away by chemical treatment, leaving the bare t-CN film. In addition, the t-CN film structurally consisting of a triazine network, as an interphase on the Cu electrode, offers homogeneously distributed lithiophilic sites, which enhance the lithium-ion transport and enable a homogeneous lithium deposition. When compared with the bare Cu foil, the lithiated t-CN interphase on the Cu exhibits a high initial Coulombic efficiency and improved cycling stability, even at high discharging rates and high lithium storage capacities. Therefore, we envision that the method herein proposed will be of high interest for developing 2D materials, in energy storage, optics, and photocatalysis, as it provides a simple solution for the synthesis of homogeneous t-CN thin films over large surfaces.

## Results and Discussion

**Figure 1:**
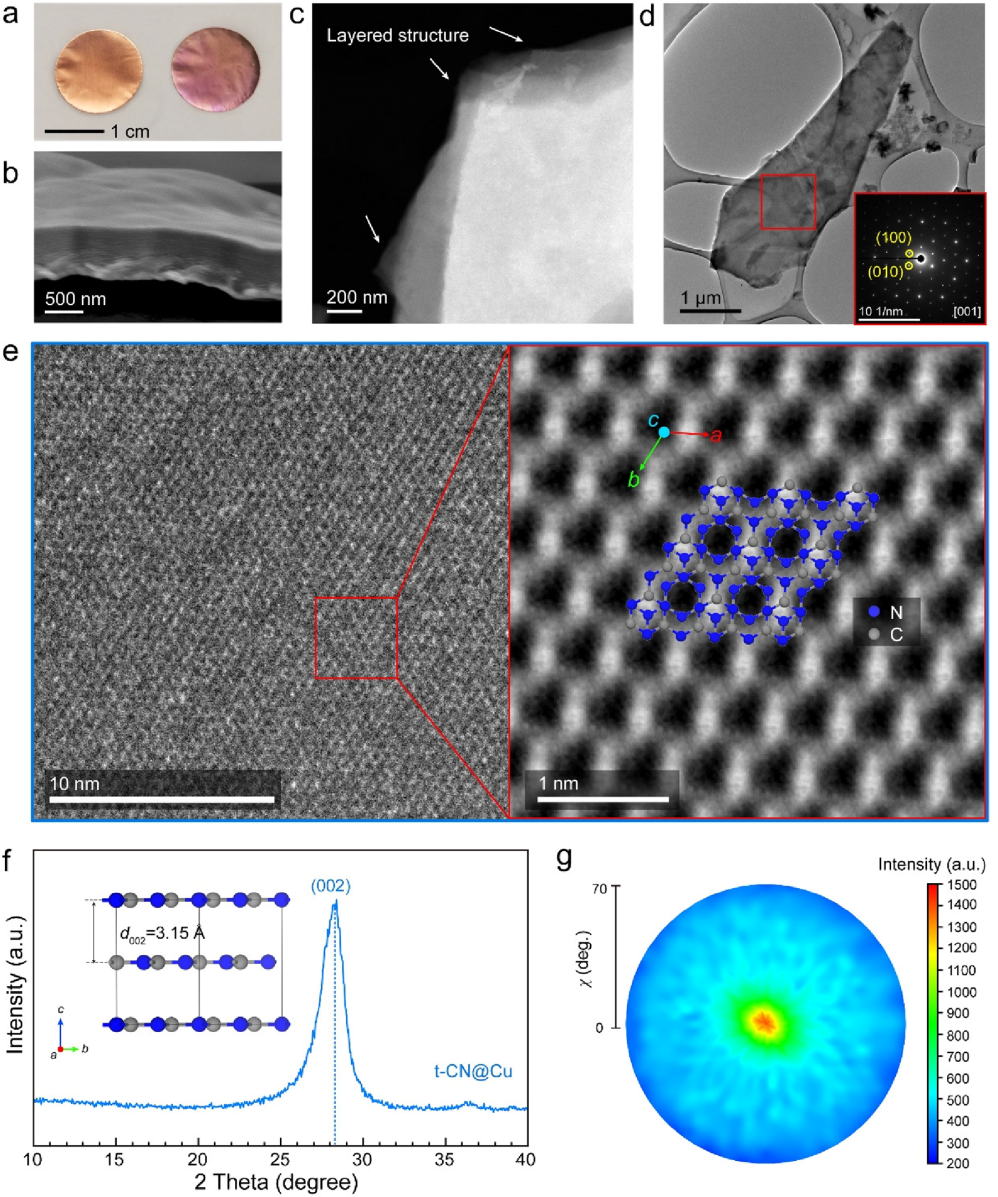
Characterization of the t-CN@Cu sample. (a) Photograph of bare Cu and t-CN@Cu electrodes with a diameter of 1.5 cm (on the left: bare Cu current collector; on the right: t-CN@Cu electrode). (b) SEM image of the cross-section view of the t-CN layer; (c) HAADF-STEM image of t-CN. (d) TEM image of t-CN thin sheet after etching the Cu substrate. Inset: Diffraction pattern corresponding to the area over the red square. (e) Left: The HR-TEM image of the t-CN thin sheet. Right: Direct visualization of the simulated CN with the triazine-based structure imposed over the experimental HRTEM image. (f) GIXRD result of t-CN@Cu. Inset: The crystallographic structure of triazine-based carbon nitride along the [210] direction. (g) Pole figure of (002) diffraction in the χ range of 0–70°.

The sample was synthesized by one-step vapor-at-metal condensation of melamine under a N_2_ atmosphere, using standard Cu foil as a target substrate (see details in the Experimental Methods). The uniform t-CN thin film on the Cu foil surface can be visually confirmed by the intense, homogeneous coloration of the substrate (Figure 1a, a punched disc from a large piece of sample). In a qualitative manner, the high-visibility interference fringes in the ultraviolet visible near-infrared (UV–vis–NIR) spectrum support that the t-CN film is homogeneous (Figure S1). At the microscale, the morphology of the coating resembles the morphology of the bare substrate as shown in atomic force microscopy (AFM) images (Figure S2), therefore pointing toward a conformal growth of the t-CN thin film over the Cu substrate.^25^ Indeed, the cross-sectional image confirms the presence of a layered material with a thickness of ca. 700 nm (Figure 1b), in good agreement with the results from the AFM (Figure S3). Scanning electron microscopy (SEM) (Figure S4) confirms that the film is highly homogeneous and crack-free over large areas, with a wavy surface that resembles that on the bare Cu substrate. At the nanoscale, the thin film material reveals a layered structure typical of 2D materials, as shown by the scanning transmission electron microscopy (STEM) image. To emphasize the presence of a layered structure, a high-angle annular dark-field (HAADF-)STEM image was collected where the stacking of 2D layers can be clearly seen by the sharply defined layers at the edge of the film (Figure 1c). The structure of the deposited t-CN was further investigated using high-resolution (HR-)TEM images and selected area electron diffraction (SAED). A highly defined diffraction pattern from a selected area (ca. 1 μm^2^) of the thin t-CN sheet (red square in Figure 1d, inset), reveals the presence of a well-defined crystalline structure, resembling the one of graphitic carbon nitride materials over a large area (Figure. 1e).^21,26^ Further investigations in other areas and samples provide similar diffraction patterns, confirming that the material grows reproducibly and homogeneously in these conditions (Figure S5). The corresponding fast Fourier transform (FFT) image shows that the in-plane *d*-spacings of (100) and (010) lattice planes are both 0.439 nm, very close to the values obtained for the theoretically predicted AB-stacking graphitic t-CN (0.437 nm) (Figure S6).^19^ In order to better visualize the structure at the nanoscale, background noise was removed by means of FFT filtering. The atomistic model of C and N from the AB-stacking triazine-based structure along the [001] zone axis is superimposed well on the experimental HRTEM image (Figure 1e, right). Furthermore, the FFT-filtered TEM image well-resembles the staggered graphite-like carbon nitride calculated by Algara-Siller et al., supporting further that the structure obtained with this method resembles the one of AB-stacking t-CN.^19^

X-ray diffraction (XRD) measurements were exploited to evaluate the orientation of the material with respect to the substrate. The t-CN thin film shows a main diffraction peak at 28.3°, as shown in Figure 1f, which is attributed to the (002) lattice plane with an interlayer distance of 0.315 nm. This (002) interlayer distance of the t-CN layer is lower than the interlayer space of graphite (0.335 nm) but consistent with the strong van der Waals’ forces between triazine rings in adjacent layers.^27^ We acknowledge that the interlayer distance is lower than the theoretically calculated value for the AB-stacking t-CN (0.329 nm), and we anticipate here a that the Cu plays a role in this.^18,19^ The pole figure of (002) diffraction (Figures 1g and S7) shows that the diffraction signal occurs only perpendicular to the surface (χ = 0°), pointing to a highly preferred stacking orientation of t-CN layers parallel to the Cu surface, in good agreement with the morphologies observed in the SEM and STEM images.

**Figure 2:**
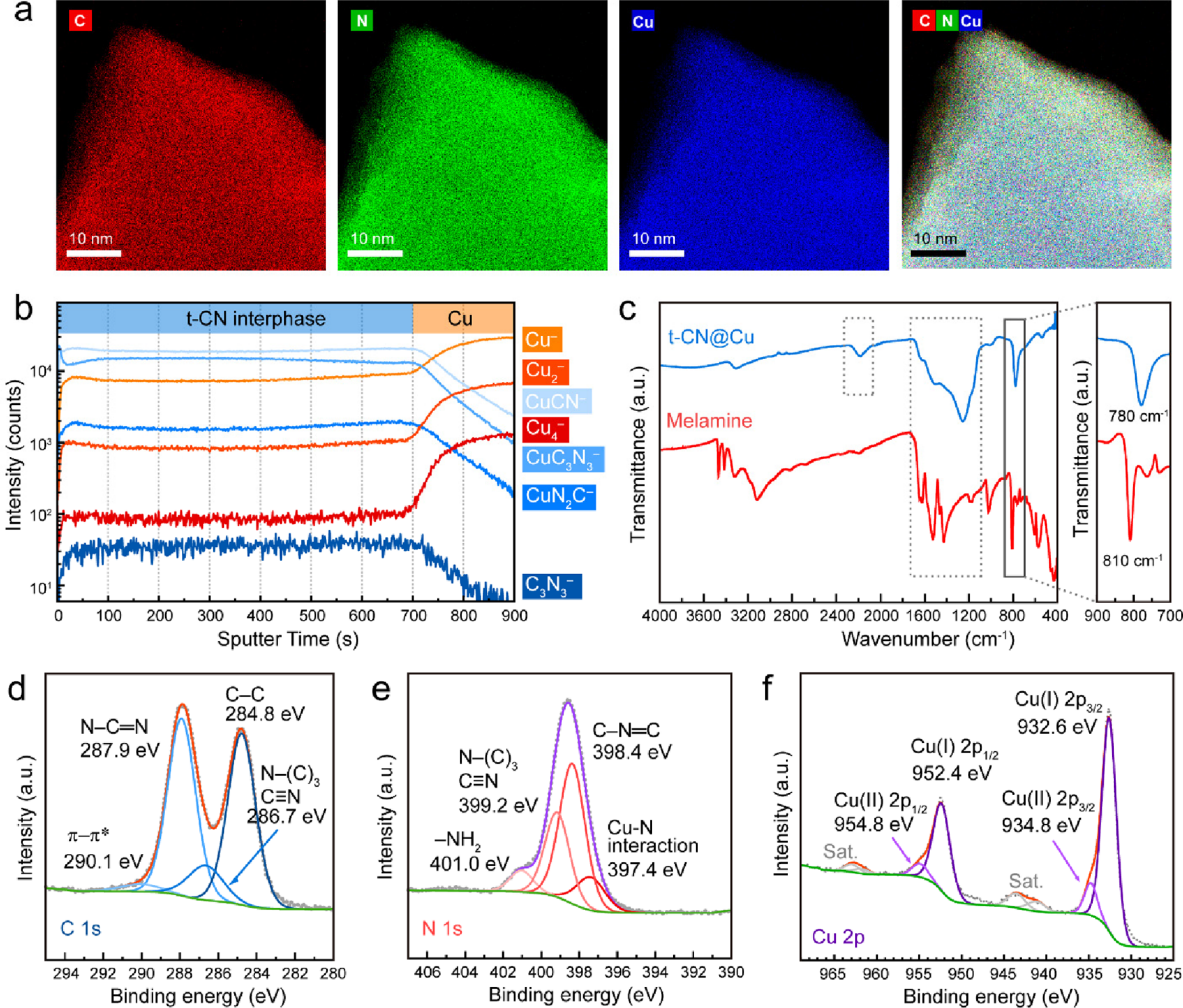
Chemical composition of the t-CN layers on Cu foil. (a) STEM-EDX elemental mapping on one piece of t-CN film without Cu substrate. (b) ToF-SIMS depth profile through the 700 nm thick t-CN layers on the top of Cu foil. (c) FT-IR spectra of t-CN@Cu and melamine, and XPS spectra of (d) C 1s, (e) N 1s, and (f) Cu 2p of the t-CN@Cu sample.

To evaluate the chemical composition and its homogeneity across the thickness of the sample, t-CN was characterized by means of spectroscopic methods, such as EDX spectroscopy (in SEM and TEM), time-of-flight secondary ion mass spectrometry (ToF-SIMS), X-ray photoelectron spectroscopy (XPS), electron energy-loss spectroscopy (EELS), and Fourier transform infrared spectroscopy (FT-IR) (Figure 2). The elemental mapping of the thin film on Cu foil detected by the energy-dispersive X-ray (EDX) spectroscopy in SEM further confirms that the method proposed leads to a high-homogeneity and crack-free film, with a homogeneous distribution of C, N, and Cu (in red, blue, and yellow, respectively, Figure S8) in the sample. However, the contribution from the Cu signal cannot be unequivocally attributed to the sample or the substrate, as the depth penetration of the X-ray is in the range or larger than the thickness of the sample. By means of chemical etching (details in the Experimental Methods section), we were able to quantitatively dissolve the Cu substrate, and the resulting free-standing t-CN film obtained was investigated by EDX in STEM mode. The collected EDX mapping of a t-CN film edge shows a homogeneous distribution across the entire area for C, N, and Cu elements (Figures 2a and S9). This points toward the formation of a 2D hybrid material containing a covalent skeleton with copper inclusions, to which we attribute the responsibility for the reduced interlayer distance observed in the material with respect to conventional carbon nitrides and theoretical calculations on t-CN structures. Indeed, ToF-SIMS depth profile analysis shows that the t-CN film is homogeneous across all of the thicknesses (Figure 2b). The selected CuC_*x*_N_*y*_ and C_*x*_N_*y*_ fragments (such as CuCN^–^, CuC_3_N_3_^–^, CuN_2_C^–^ , and C_3_N_3_^–^) signals remain constant throughout the film thickness and deplete upon reaching the Cu substrate (after 700 s sputter time), finding good agreement with our previous thickness measurements. Furthermore, the 3D overlay figures further reveal the presence of homogeneously distributed Cu_*z*_C_*x*_N_*y*_ fragments across the whole sample thickness (Figure S10). Typical contaminations, such as oxygen-containing fragments, were not detectable in our analyses. The arising of several CuC_*x*_N_*y*_ fragments points toward the presence of single Cu atoms homogeneously dispersed in a C_*x*_N_*y*_ matrix and shows that these Cu atoms are stabilized by the nitrogen atoms in the t-CN plane. The C- and N-containing fragments observed in the Tof-SIMS spectrum of t-CN@Cu sample are different from heptazine-based carbon nitride materials (Figure S11).^28^ We expect that these Cu species are involved in the formation of the t-CN structure and potentially play a role in stabilizing it. Theoretical calculations made on graphitic t-CN structure reveal that, in t-CN, the repulsion between the nitrogen lone pairs of the triazine units in the molecular planes leads to corrugation of the structure, which we have not noticed in our sample when scanning tenths of areas in TEM mode.^18^

The FT-IR spectrum of t-CN@Cu (Figure 2c) shows intense characteristic peaks in the fingerprint region (1100–1800 cm^–1^) that are ascribed to the C–N vibrations of the condensed triazine-based structure. Notably, the low signal in the range 2400–4000 cm^–1^ confirms a minor contribution from H-containing groups such as, −NH– and −NH_2_.^17,29^ The N≡C stretching peak at 2180 cm^–1^ correlates with the presence of nitrile edge terminations of the layers.^30,31^ The characteristic band attributed to the out-of-plane bending mode of the triazine ring in melamine is found to be significantly red-shifted and broadened in the t-CN sample (from 810 to 780 cm^–1^). Similar to the cases of metal single atoms@N-doped carbon, we attribute this shift to the polarization of charge distribution arising from the coordination between the triazinic N and Cu atoms.^32^ In this perspective, coordinated Cu is stabilized in the structural pores by the N of the triazine units during the condensation of the t-CN structure. The distance between the central point of the triangular void and the closest N atoms is about 1.65 Å, which is similar to the distance of the first shell of Cu–N coordination in Cu single-atom materials.^33−35^ Graphitic carbon nitride materials have been reported to serve as hosts supporting single metal atoms or geminal atoms.^33,36^ The shift in FT-IR and fragments in ToF-SIMS could be translated to the homogeneous distribution of Cu atoms within the covalent structure of t-CN. In the AB-staking arrangement previously depicted, the Cu atoms are located in the t-CN trigonal voids and between the tertiary nitrogen atoms of the layers in the immediate proximity, which we expect to partially contribute to the stabilization of the Cu atoms and thus reducing the interlayer spacing.

The existence of a highly conjugated network is confirmed by the presence of intense *sp*^*2*^ N–C=N (287.9 eV) and C–N=C (398.4 eV) peaks in the XPS C 1s and N 1s spectra, respectively (Figure 2d,e). The N 1s peak at 399.4 eV is attributed to the bridging N–(C)_3_ connecting the triazine rings to their neighboring units. The XPS results are consistent with the electron energy-loss spectroscopy (EELS) spectra, showing very similar features of the C-K and N-K edges, especially with sharp π* transition peaks, typical of highly conjugated frameworks (Figure S12).^37^ The C/N atomic ratios are 0.77 and 0.78 as calculated from EELS and XPS, very close to the ideal value of 0.75, respectively (Figures S12 and S13 and Tables S1 and S2). The weak signal at 401.0 eV in the N 1s spectrum is attributed to the H-containing terminal groups (e.g., −NH_2_) on the surface of the t-CN layer. (Figure 2e). The presence of Cu in the as-synthesized t-CN layers is further confirmed by a Cu 2p XPS signal, displaying two peaks typically attributed to two different oxidation states, Cu(I) and Cu(II) (Figure 2f), resembling the signal of Cu single atoms in a heptazine-based carbon nitride matrix.^33,38^ The atomic ratio of Cu/N sums to about 0.36 as calculated from the XPS spectrum (Figure S13, Table S2).

In the thermal vapor deposition process, the semiclosed synthesis environment involves a higher partial pressure of the intermediates, such as cyanamide, and byproducts, such as ammonia, in the crucible. The Cu foil does not only serve as a substrate, but during the reaction, the Cu atoms diffuse from the substrate through the material, stabilizing the t-CN structure and preventing formation of heptazine rings via the triazine ring-opening and -closing pathway.^39^ The computational model showed that the heptazine unit is more thermodynamically stable than triazine in the graphitic phase under ambient conditions. Therefore, a triazine-based structure has been rarely obtained from commonly used precursors via a solid-state synthesis. Kessler and Schnick found the transformation from heptazine to triazine in a closed system during the ionothermal process.^40^ In this case, the molten salts provide a solvation effect to depolymerize the heptazine-based unit, followed with intercalation of Li^+^ and X^–^ ions to form stable triazine-based frameworks.^41^ It is worth noting that the radii of copper ions (Cu^+^: 0.77 Å; Cu^2+^: 0.73 Å) are very close to those of lithium ions (0.76 Å). In the thermal vapor deposition process, the Cu atoms, which are anchored by N sites (e.g., N in heteroatom rings, bridging N, or cyano groups), can act in a similar role of suppressing the formation of heptazine from triazine-based units and other small-molecule intermediates (e.g., ammonia, cyanamide, dicyandiamide, etc.). As a result, a highly crystalline triazine-based graphitic phase grows at the gas/Cu interface at 500 °C, lower than the usually applied temperature for the formation of heptazine-based carbon nitrides on nonmetallic substrates.^37^ Indeed, according to thermogravimetric analysis coupled with mass spectrometry (TGA-MS), in the presence of Cu and melamine, we noticed a sharp NH_3_ release occurs at 450 °C, which is the byproduct of the condensation reaction of melamine (Figure S14). However, this temperature is too low for the formation of the crystalline t-CN phase, while we found indications of the depolymerization of t-CN when the synthesis temperature is raised to 550 °C (Figure S15).

Small structural pores, a high degree of crystallinity, and homogeneity are expected to hinder the diffusion of the solvent molecules through the t-CN film, thus leading to an increase in the ionic transport performances and stability of the electrode. Therefore, the t-CN deposited on Cu foil (t-CN@Cu) was investigated as an anode electrode for Li metal batteries. At first, an activation process was performed at a constant current density of 0.1 mA cm^–2^ (Figure S16a). The presence of abundant N-containing structures provides Lewis-base-like sites that can give rise to well-distributed Li–N interactions upon initial lithiation.^42−44^ The EIS results reveal that the lithiated lithiates have a t-CN reduced interfacial impedance with respect to the native SEI on the bare Cu current collector (Figure S16b). According to the results of the galvanostatic intermittent titration technique (GITT), the activated t-CN layer possesses a high chemical Li^+^ diffusion coefficient (*D*_Li_^*+*^) of 10^–12^∼10^–10^ cm^2^ s^–1^ at different states-of-charge (SOCs) during activation (Figure S17). This is close to that of common solid-state electrolytes and higher than most of electrode materials usually reported.^45−49^ Therefore, the well-organized Li^+^ transport channels ensure a high Li^+^ conductivity across the t-CN layer, which enables a fast Li^+^ transport kinetics and prevents excessive interfacial concentration polarization. Due to the semiconductive nature of carbon nitrides, the t-CN@Cu electrode shows a slightly higher Ohmic resistance than bare Cu, which is essential to suppress the electrical contact with the solvent and, eventually, avoid solvent decomposition.

**Figure 3:**
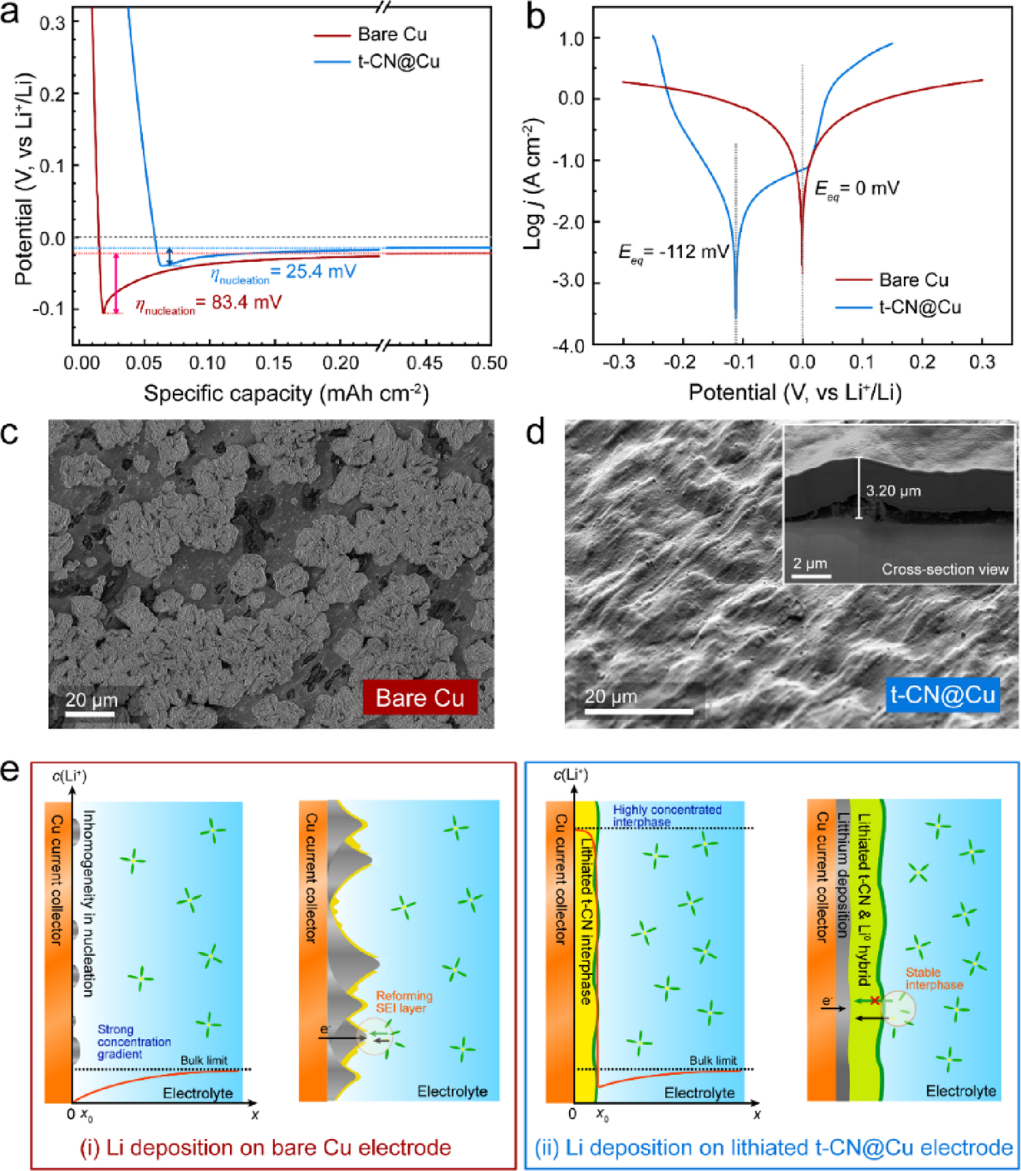
Comparison of electrochemical behaviors of bare Cu and t-CN@Cu electrodes. (a) The Li deposition test of bare Cu and t-CN@Cu electrodes at a constant current density of 0.2 mA cm^–2^ with a specific capacity of 0.5 mAh cm^–2^. (b) Tafel curves of bare Cu and t-CN@Cu electrodes after activation cycles. SEM images of (c) bare Cu and (d) t-CN@Cu electrodes after 0.5 mAh cm^–2^ of Li deposition (inset: FIB-SEM images and elemental mappings of the cross-section view of t-CN@Cu electrode after 0.5 mAh cm^–2^ Li deposition. (e) The illustration of Li deposition behavior on (i) bare Cu and (ii) lithiated t-CN@Cu electrodes.

To explore the Li deposition behavior on the activated samples, galvanostatic charge-discharge (GCD) and Tafel curves were measured in a half-cell system against a Li metal electrode. The nucleation overpotential (η_nucleation_) of the t-CN@Cu electrode (25.4 mV) is significantly lower than that of bare Cu (83.4 mV) at a current density of 0.2 mA cm^–2^ and a slightly lower growth overpotential of the t-CN@Cu electrode (Figure 3a). After the performance of CN@Cu samples prepared at different temperatures was investigated, the t-CN@Cu prepared at 500 °C was selected as the optimal sample for the following study (Figures S18–20). Unlike the bare Cu electrode, the anodic Tafel curve of the t-CN@Cu electrode shows an additional stage, indicating a change in the Li storage behavior (Figure 3b). The negative shift of the equilibrium potential in the t-CN@Cu electrode reflects the stabilization effect of the t-CN layer on metallic Li, which is advantageous for achieving primary nucleation uniformly distributed on the electrode. Li et al. reported a defective C–N coating film as an artificial SEI layer on Cu for homogenizing the Li plating behavior.^15^ In this work, an additional passivation area is observed in the Tafel curve, confirming the uniform coverage of the condensed t-CN interphase. To the best of our knowledge, there were no similar phenomena reported in previous studies using electrodes coated by condensed carbon-nitride-based materials.^50^ Distinguished from granular material coating layers, the homogeneous and crack-free t-CN film as the interphase shows the improved behavior of Li^+^ transport and storage within this 2D triazine-based layer.^14,15,51^

At the microscale, the Li deposition on the bare Cu foil displays a dendritic, island-like growth of lithium metal (Figure 3c). On the other hand, the surface of t-CN@Cu electrode, in the same conditions, reveals a morphology similar to the pristine t-CN film (Figure S4b), indicating a uniform Li electrodeposition within or beneath the coating interphase (Figure 3d). The cross-sectional morphology of lithium deposition on the t-CN@Cu electrode was determined using focused ion beam (FIB)-SEM (Figure 3d, inset). The total thickness of the electrode after the Li deposition, at a capacity of 0.5 mAh cm^–2^ increases by approximately 2.00 μm compared to the electrode after initial lithiation (∼1.20 μm, Figure S21) and exhibits a lower volume change compared to the Li metal anode plating with the same areal capacity (∼2.45 μm). This finding suggests that a highly dense, structurally protected, and thus safe lithium deposition was achieved by means of the t-CN layer. The EDX elemental mapping shows O-containing species aggregated exclusively on the outer surface of t-CN layers, confirming that the thickness increase was not due to swelling by solvent uptake but rather lithium deposition (Figure S22). After the delithiation process (discharged to 2.0 V vs Li^+^/Li), the t-CN layer retains the homogeneous morphology with a reduced thickness, demonstrating the reversibility and high mechanical stability of the lithium deposition and stripping cycle (Figure S23).

Based on the electrochemical Li deposition mechanism, a schematic diagram is illustrated to depict the function of the t-CN interphase on the Cu electrode (Figure 3e). The reference bare Cu electrode has a weak affinity for lithium, which leads to inconsistencies in interfacial charge transport and initial lithium nucleation. These heterogeneities are then enhanced during cycling, with dendrites growing toward the bulk of the electrolyte and fracturing the solid electrolyte interphase (SEI), leading to an accelerated battery degradation (Figure 3e-i).^52^ The lithiated t-CN provides a localized, highly concentrated interphase (Figure S24), which diminishes the interfacial concentration gradient, enabling a highly dense Li deposition at the interface with the Cu foil (Figure 3e-ii).

**Figure 4:**
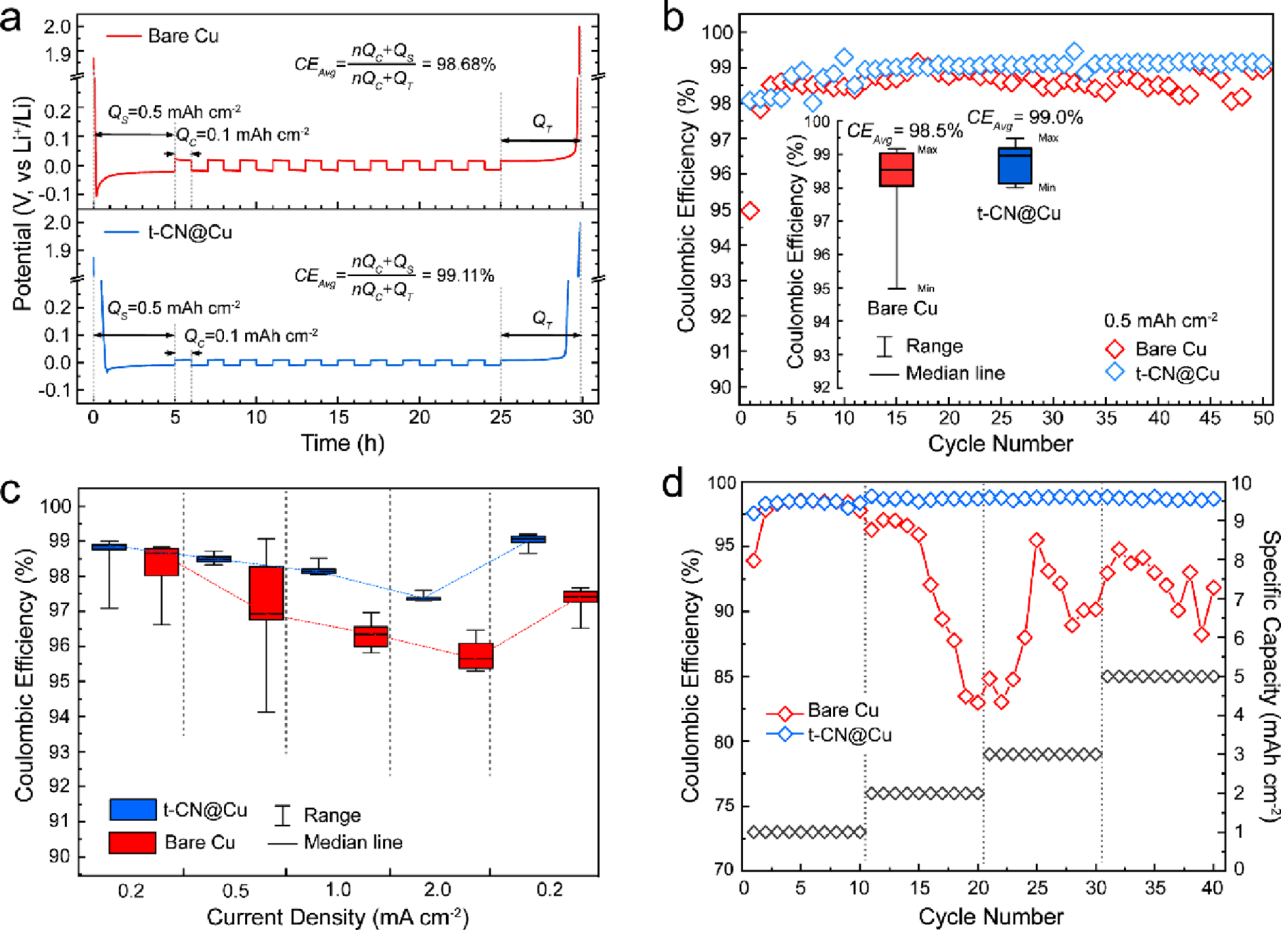
Comparison on electrochemical performances between bare Cu and t-CN@Cu electrodes. (a) Average Coulombic efficiencies of bare Cu and t-CN@Cu electrodes. (b) Cycling performance of Cu||Li and t-CN@Cu||Li cells at the current density of 0.2 mA cm^–2^. Inset: Box chart plot of the Coulombic efficiency distribution during cycling. (c) Rate performance of Cu||Li and t-CN@Cu||Li cells with a capacity of 0.5 mAh cm^–2^ at various discharge current densities of 0.2, 0.5, 1.0, and 2.0 mA cm^–2^. (d) Performances of Cu||Li and t-CN@Cu||Li cells with various specific capacities of 1, 2, 3, and 5 mAh cm^–2^ at a current density of 0.5 mA cm^–2^.

The cycling performance of the t-CN@Cu electrode was evaluated using a half-cell configuration with lithium metal serving as both the reference and counter electrode with 1 M LiTFSI in dimethoxyethane/dioxolane (DME/DOL) as electrolyte. Initially, compared to a bare Cu current collector, the t-CN@Cu electrode displays a higher average Coulombic efficiency (CE_Avg_) of 99.11% and a lower polarization voltage of only 17.6 mV during the deposition/stripping processes (Figure 4a). The lithiated t-CN as a highly concentrated interphase homogenizes the interfacial concentration gradient, which can decrease the concentration polarization. In addition, the anchored Cu atoms change the electrostatic potentials around the nearby triazine-based networks, which can increase the lithiophilicity of the N sites to improve the Li affinity and decrease the polarization potential during Li deposition processes.^53^ At a moderate current density of 0.2 mA cm^–1^, the bare Cu current collector demonstrates a low initial CE (∼95%) and a relatively dispersed distribution of CEs when cycling at a 0.5 mAh cm^–2^ specific capacity. The lithiated t-CN@Cu electrode exhibits higher cycling stability with a CE_Avg_ of 99.0% with an initial CE of >98% for 50 cycles (Figure 4b). The formation of dendrites and “dead Li” typically worsens at higher current densities due to increased interfacial concentration polarization effects. The lithiated t-CN@Cu electrode demonstrates significantly higher stability than the bare Cu electrode, even at higher current densities. Notably, when the current density returned from 2 to 0.2 mA cm^–2^, the CEs of bare Cu drop from ∼98.5 to ∼97.3%, while the CEs of t-CN@Cu electrode are recovered to original or even higher values, implying that the t-CN interphase is stable or even improves the Li^+^ transport (Figures 4c and S25). The cycling stability of the current collector based on multiple Li deposition/stripping processes is of paramount importance to achieve the high-energy density targets. For this purpose, the specific areal capacity was increased stepwise from 1 to 5 mAh cm^–2^, which meets the capacity demands of high-loading cathodes applied in practical full batteries. The lithiated t-CN@Cu electrode displays a high CE_Avg_ of about 98.7% with a high capacity of 5 mAh cm^–2^ and an increased stable cyclability compared to that of a bare Cu electrode (Figures 4d, S26). In addition, compared with bare Cu electrode, the improved cycling stability and lower potential polarization are observed in the t-CN@Cu electrode with a cycling capacity of 1.0 mAh cm^–2^ at the current density of 0.5 mA cm^–2^ after the multicapacity cycling tests (Figure S27). Furthermore, full-cell tests were investigated with LiFePO_4_ as the cathode material (Figure S28). The full cell based on a lithiated t-CN@Cu anode showed much better stability than that using bare Cu. These results show the high potential for application of the t-CN@Cu as an electrode for lithium metal batteries.

## Conclusions

In this work, we introduced a simple and feasible synthesis method for triazine-based graphitic carbon nitride films. The interfacial polycondensation of melamine on the Cu metal surface in a semiclosed system leads to the formation of a highly condensed, homogeneous graphitic phase at a relatively low temperature (around 500 °C). The obtained t-CN film was confirmed showing a similar in-plane crystalline structure with calculated AB-stacking triazine-based graphitic carbon nitride as observed in TEM. The vacuum-metal interfacial reaction mechanism induced an oriented growth of the 2D triazine-based layers parallel to the Cu metal surface as shown in SEM images and the pole figure of XRD. The homogeneity of the t-CN film throughout its 700 nm thickness was confirmed by ToF-SIMS and the interference in the UV–vis–NIR spectrum. Cu atoms homogeneously dispersed in the t-CN phase, coordinated with triazine-N in the structural pores, potentially suppress the formation of the heptazine structure. In summary, we are able to produce homogeneous triazine-based carbon nitride films on commercial Cu foil on a centimeter scale.

Our work demonstrated the application of this t-CN@Cu sample as an anode for Li metal batteries. The activated t-CN film with a N-rich composition and defined structural pores provides abundant lithiophilic sites and homogeneous charge transport as a highly concentrated interphase between the Cu electrode and liquid electrolyte. This interfacial engineering approach improves the uniformity of dense electrochemical Li deposition on the Cu current collector and weakens the solvent decomposition, thus setting the basis for an improved cycling performance.

As the prospects of this work, the developed vapor-metal interfacial condensation method is applicable for effectively producing high-quality, large-area 2D triazine-based graphitic carbon nitride materials on a planar substrate. The confirmed triazine-based structure provides opportunities to establish clear structure-property relationships of these carbon nitride materials, showing the potential of applications such as energy conversion, membrane separation, and optical applications. The geometric sizes of t-CN films are tunable via optimizing the parameters of the synthesis condition. The metal substrates are feasible to be removed by chemical methods, leaving self-standing t-CN films, which allows transferring the films to other substrates. Further ongoing projects are aiming to extend the synthesis strategy and applications of these materials.

## Experimental Methods

### Material Preparation

The t-CN@Cu is prepared by thermal vapor deposition in a corundum crucible forming a semiclosed system in N_2_ atmosphere. In detail, 200 mg of melamine (Sigma-Aldrich, 99%) was used as the precursor put at the bottom of an alumina crucible (99.7%, Al_2_O_3_; Φ_Top_: 63 mm; height: 54 mm; volume: 100 mL). A commercial battery-grade Cu foil (PI-KEM, Ltd., Cu content >99.95%) was first immersed in 0.5 M HCl aqueous solution for few minutes and then washed by deionized water and ethanol three times. The obtained clean Cu foil was used to cover the crucible on the top as the substrate. The assembled thermal vapor deposition setup was heated at a rate of 150 °C/h up to a certain condensation temperature (400, 450, 500, and 550 °C), and the temperature was then held for 4 h to obtain the product.

### Material Characterization

The morphologies of as-prepared t-CN@Cu samples were investigated by SEM using Gemini 1550 Zeiss AG at an acceleration voltage of 3 kV. The high-angle annular dark-field scanning TEM (HAADF-STEM) images and electron energy-loss spectroscopy (EELS) were conducted with a JEOL-ARM200F transmission electron microscope with a Gatan image filter (GIF), at 80 kV with 10 μA emission current. An EELS spectrum was taken with energy dispersion of 0.25 eV/Ch. EELS data was processed by a Gatan digital micrograph (GMS 3). For high-resolution (HR-)TEM measurement, a piece of t-CN@Cu was first immersed in 0.1 M aqueous solution of ammonium persulfate (Sigma-Aldrich, >98%) to etch the Cu foil. When Cu was removed, the self-standing t-CN film was washed and exfoliated into thin sheets by ultrasonic dispersion in ethanol. Then, the TEM sample was obtained by dispersing thin sheets on a gold TEM grid with lacey carbon support and drying on air. The signals are extracted after zero loss peak (ZLP) calibration and plural scattering deconvolution for better visualization. EELS data processing has been done using a Digital Micrograph 3.4 software package. Analysis of the morphology and elemental composition of the cross-section of samples were carried out by performing trenches using a Zeiss Crossbeam 540 Focused Ion Beam-Scanning Electron Microscopy (FIB-SEM) (Zeiss Microscopy GmbH, Oberkochen, Germany). The samples were elevated to the height of 5.1 mm, which corresponds to the coincident point of the two beams and tilted to 54°. Trenches of approximately of 70 μm in width and 30 μm in length were milled at 15 nA. The exposed surface was polished and imaged with a lower ion beam current (700 pA). The electron beam was then focused on the polished exposed surface at 1.5 keV and 500 pA. The energy-dispersive X-ray spectroscopy (EDS) microanalysis was performed on the tilted exposed surfaces with an Oxford X-Max 100 mm^2^ at a voltage of 5 kV and a working distance of 7 mm. The ToF-SIMS (ION-TOF V GmbH) was operated with a Bi_3_^+^ primary ion gun at 25 keV and 0.2 pA beam current, with a 5 μm spot size on a 100 × 100 μm field of view, with 128 × 128 pixels rastered in sawtooth mode for 900 s of sputter time (100 μs of cycle time). The sputter gun was Cs 1 kV with 60 nA at 500 × 500 μm, noninterlaced sputtered (10 frames sputter, 1 s analysis, 1 s pause). Data was analyzed on SurfaceLab 7. Negative spectra were calibrated to C_1_^–^, O_1_^–^, C_2_^–^, C_5_^–^, C_8_^–^. Deviation remained <100 ppm for all assigned peaks. The TGA-MS measurement was performed using a thermal microbalance (Netzth TG 209 F1 Libra) coupled with a Thermostar Mass Spectrometer (Pfeiffer Vacuum) with an ionization energy of 75 eV. A small disc-shaped Cu foil matching the inner diameter of the thermogravimetric crucible was settled inside exactly, covering the top of melamine powders for TGA-MS measurement. AFM images were acquired using a commercial AFM system (NanoScope-Multimode III) in tapping mode with an Arrow NCR tip (42 N m^–1^, 285 Hz). The samples for AFM were prepared on a silicon wafer. The AFM images were only subjected to the primary first other flattening correction to remove sample tilt. FT-IR spectroscopy was performed by using a Thermo Scientific Nicolet iD5 spectrometer with an attenuated total reflection (ATR) sampling technique. The surficial chemical state was detected by XPS using CISSY equipment (Helmholtz-Zentrum Berlin, Germany) with a SPECS XR 50 Mg Kα gun and combined lens analyzer module (CLAM). The general XRD measurements and pole figure at (002) lattice plane reflection were carried out on a SmartLab (Rigaku Co., Japan) equipped with Cu Kα radiation (λ = 1.54 Å).

### Cell Assembly

The coin cells (CR2032) and Swagelok-type cells were assembled in an argon-filled glovebox. The obtained t-CN@Cu was punched and directly used as an electrode. The electrolyte was 1 M lithium bis(trifluoromethanesulfonyl)imide (LiTFSI) in 50/50 (v/v) dimethoxyethane/dioxolane (DME/DOL) (E-Lyte, GmbH). Sandwiched polypropylene-polyethylene-polypropylene (Celgard 2325) was utilized as the separator. One lithium metal chip (China Energy Lithium Co., Ltd., Li content >99.9%) was used as the counter electrode in a two-electrode cell for half-cell tests. In a three-electrode Swagelok-type cell, another lithium metal electrode was applied as an individual reference electrode. The full-cell tests were conducted in Swagelok cells by using LiFePO_4_ (LFP) as the cathode material and the same electrolyte for half-cell tests. The LFP cathode slurry was prepared by mixing active material with the conductive carbon (Super P, Alfa Aesar) and polyvinylidene difluoride (PVDF, Kynar HSV-900) binder at a weight ratio of 8:1:1 in *N*-methyl-2-pyrrolidone (NMP, Sigma-Aldrich). The obtained slurry was then coated on aluminum foil with a mass loading of about 2.75 mg cm^–2^. The specific capacity and current density in full-cell measurements were calculated by the mass of cathode active material.

### Electrochemical Testing

The electrochemical characterizations were conducted using the Biologic MPG-2 Battery Testing System at room temperature. Galvanostatic cycling tests were measured in two-electrode cells, first discharged-charged at a constant current density of 0.1 mA cm^–2^ within a voltage window of 2.0 to 0.0 V (vs Li^+^/Li) for five initial formation cycles and then discharged at a certain content current density (i.e., 0.2, 0.5, 1.0, or 2.0 mA cm^–2^) under constant capacity conditions (i.e., 0.5, 1.0, 2.0, or 5.0 mAh cm^–2^) in subsequent cycles. The electrochemical impedance spectroscopy (EIS) was recorded by using three-electrode cells in a frequency range from 100 mHz to 100 kHz. The ex situ EIS curves of the t-CN@Cu electrode were collected at various selected SOCs during the first formation cycle. The galvanostatic intermittent titration technique (GITT) was measured with a polarization process consisting of a current pulse of 0.1 mA cm^–2^ for 10 min followed by an open circuit stand process for 60 min to relax to quasiequilibrium potential. The detailed calculation of Li^+^ chemical diffusion coefficients is shown in Supporting Information. The Tafel plots were obtained on a Gamry Interface 1000 electrochemical workstation at a scan rate of 0.1 mV s^–1^ between −0.3 and 0.3 V (vs Li^+^/Li).

## Supporting Information Available

UV–vis–NIR absorbance spectrum of t-CN@Cu; AFM images and roughness of bare Cu substrate, t-CN@Cu sample, and t-CN thin film; SEM images of bare Cu substrate and t-CN@Cu sample; HR-TEM and SAED images of different t-CN sheets; HR-TEM image of t-CN sheet with corresponding FFT image of the selected region; Pole figure plots of (002) diffraction of t-CN@Cu sample; STEM image and corresponding spectra of selected regions; ToF-SIMS spectrum of t-CN@Cu sample; Three-dimensional overlay figure of CuN_*x*_C_*y*_ fragments; ADF-STEM image and corresponding EELS spectrum of the t-CN layer; XPS spectrum of t-CN@Cu; TGA-MS results of melamine; SEM images and XPS spectra of C 1s and N 1s of CN@Cu samples prepared at different temperatures; Comparison of FT-IR spectra of precursor and samples prepared at different condensation temperatures; Electrochemical activation of the t-CN@Cu electrode; Comparison of electrochemical Li storage behavior of CN@Cu electrodes prepared at different temperatures; GITT measurements of t-CN@Cu electrode; FIB-SEM images of the cross-section view of activated t-CN@Cu; EDX mapping of the cross-section view of t-CN@Cu electrode after 0.5 mAh cm^–2^ Li deposition; FIB-SEM images of the cross-section view of t-CN@Cu electrodes after Li stripping; Activation of t-CN@Cu electrode; Galvanostatic charge/discharge curves of bare Cu and t-CN@Cu electrodes at various discharging current densities; Galvanostatic charge/discharge curves of bare Cu and t-CN@Cu electrodes with various specific areal capacities; Cycling performance of bare Cu||Li and t-CN@Cu||Li cells after the multicapacity cycling tests; Full-cell performance of LFP||Cu and LFP||t-CN@Cu batteries (PDF)
