# Supplementary material for: Triazine-Based Graphitic Carbon Nitride Thin Film as a Homogeneous Interphase for Lithium Storage
Source: ACS Nano. 2024 Jan 9;18(3):2066–76. doi: 10.1021/acsnano.3c08771 (PMC10811665; doi:10.1021/acsnano.3c08771)
Supplement: Supplementary file 1 — nn3c08771_si_001.pdf [file nn3c08771_si_001.pdf]

Supporting Information

Triazine-based Graphitic Garbon Nitride Thin Film as a Homogeneous Interphase for Lithium Storage

Zihan Song,[a*] *Jing Hou,*[a] *Emeline Raguin,*[b] *Angus Pedersen,*[c, d] *Enis Oğuzhan Eren,*[a] *Evgeny Senokos,*[a] *Nadezda V. Tarakina,*[a] *Paolo Giusto,*[a*] *and Markus Antonietti* [a]

[a] Colloid Chemistry Department, Max Planck Institute of Colloids and Interfaces, Am Mühlenberg 1, Potsdam 14476, Germany
[b] Biomaterials Department, Max Planck Institute of Colloids and Interfaces, Am Mühlenberg 1, Potsdam 14476, Germany
[c] Department of Chemical Engineering, Imperial College London, SW7 2AZ, London, UK
[d] Department of Materials, Imperial College London, SW7 2AZ, London, UK

*e-mail: Zihan.Song@mpikg.mpg.de; Paolo.Giusto@mpikg.mpg.de

Contents

Figure S1 | UV-vis-NIR absorbance spectrum of t-CN@Cu
Figure S2 | AFM images and roughness of bare Cu substrates and t-CN@Cu samples
Figure S3 | AFM analysis of t-CN film
Figure S4 | SEM images of bare Cu substrate and t-CN@Cu sample
Figure S5 | HR-TEM and SAED images of different t-CN sheets
Figure S6 | HR-TEM image of t-CN sheet with corresponding FFT image of the selected region
Figure S7 | Pole figure plots of (002) diffraction of t-CN@Cu sample
Figure S8 | SEM image and EDX mapping of t-CN@Cu sample
Figure S9 | The STEM image and corresponding spectra of selected regions
Figure S10 | ToF-SIMS spectrum of t-CN@Cu sample
Figure S11 | Three-dimensional overlay figure of CuN_xC_y fragments
Figure S12 | ADF-STEM image and corresponding EELS spectrum of the t-CN layer
Figure S13 | The XPS spectrum of t-CN@Cu
Figure S14 | TGA-MS results of melamine
Figure S15 | The comparison of FT-IR spectra of precursor and samples prepared at different condensation temperatures
Figure S16 | The electrochemical activation of the t-CN@Cu electrode
Figure S17 | GITT measurements of t-CN@Cu electrode
Figure S18 | SEM images of CN@Cu samples prepared at different temperatures
Figure S19 | XPS spectra of C 1s and N 1s of CN@Cu samples prepared at different temperatures
Figure S20 | The comparison of electrochemical Li storage behavior of CN@Cu electrodes prepared at different temperatures
Figure S21 | FIB-SEM images of the cross-section view of activated t-CN@Cu
Figure S22 | EDX mapping of the cross-section view of t-CN@Cu electrode after 0.5 mAh cm^{-2} Li deposition
Figure S23 | FIB-SEM images of the cross-section view of t-CN@Cu electrodes after Li stripping
Figure S24 | The activation of t-CN@Cu electrode
Figure S25 | Galvanostatic charge/discharge curves of bare Cu and t-CN@Cu electrodes at various discharging current densities
Figure S26 | Galvanostatic charge/discharge curves of bare Cu and t-CN@Cu electrodes with various specific areal capacities
Figure S27 | The cycling performance of bare Cu||Li and t-CN@Cu||Li cells after the multi-capacity cycling tests.
Figure S28 | Full cell performance of LFP||Cu and LFP||t-CN@Cu batteries.

Table 1 | EELS analysis of the spectrum in Figure S9b

Table 2 | Atomic ratio on surface (by XPS) for t-CN@Cu sample

Reference

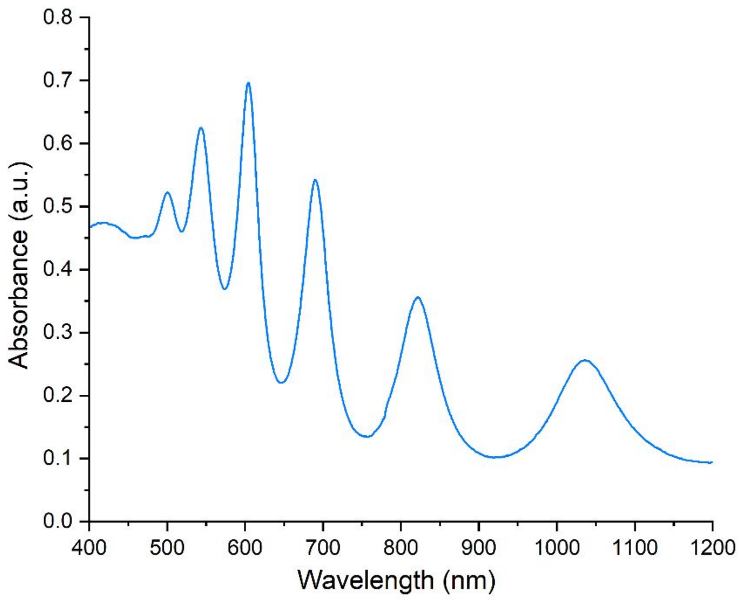

Figure S1 | UV-vis-NIR absorbance spectrum of t-CN@Cu.

Note:

The multiple oscillations in the wavelength range of 400-1200 nm are interference fringes due to the constructive and destructive interferences of various internally reflected wavelengths of light inside t-CN layer. This phenomenon confirms the parallel orientation of t-CN layer stacking and further represents the homogeneity of the t-CN phase throughout the thickness.

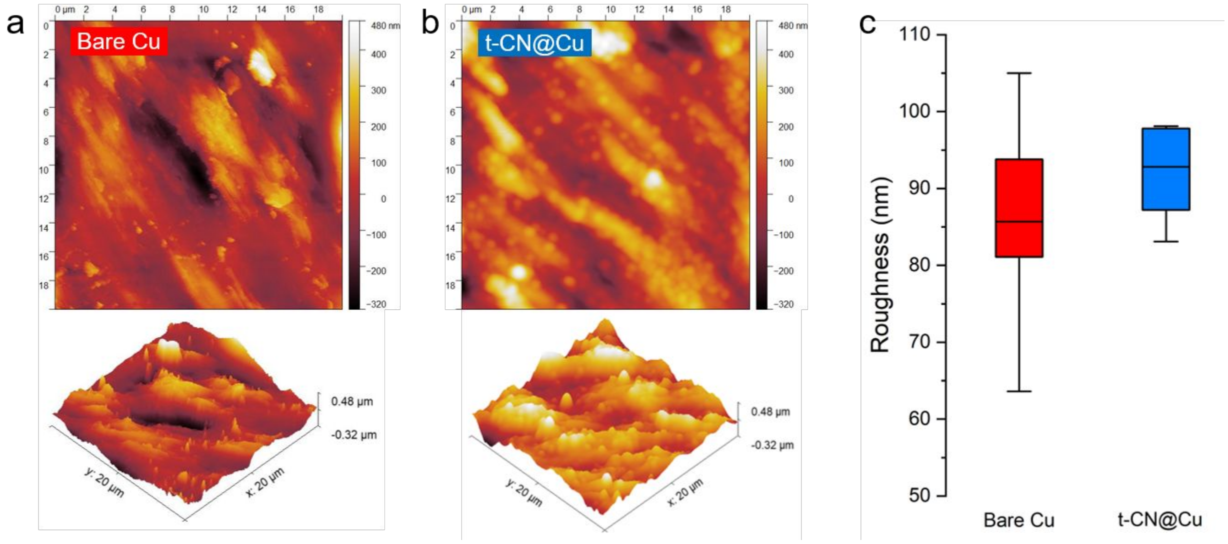

Figure S2 | AFM images and roughness of bare Cu substrates and t-CN@Cu samples. The AFM image and 3D version of (a) bare Cu substrate and (b) t-CN@Cu sample; (c) The roughness statistics of six samples of bare Cu and t-CN@Cu each (box: 25~75%).

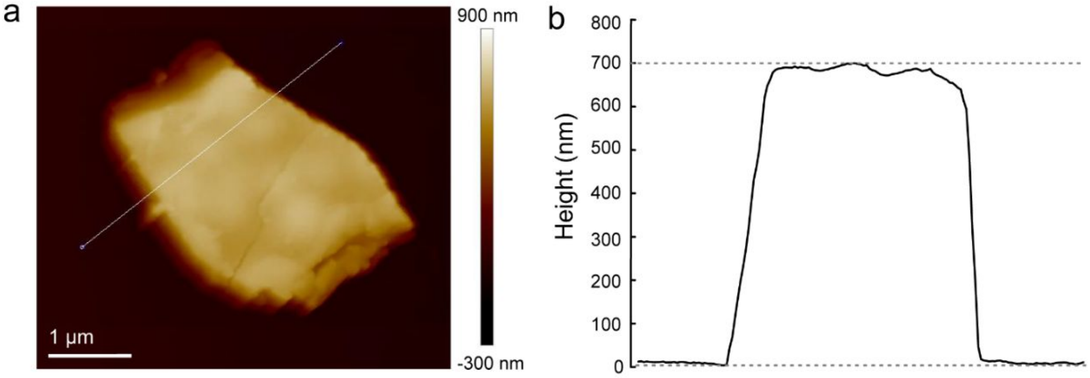

Figure S3 | AFM analysis of t-CN film. (a) The AFM image of a piece of t-CN sheet and (b) the height plot of selected cutting line.

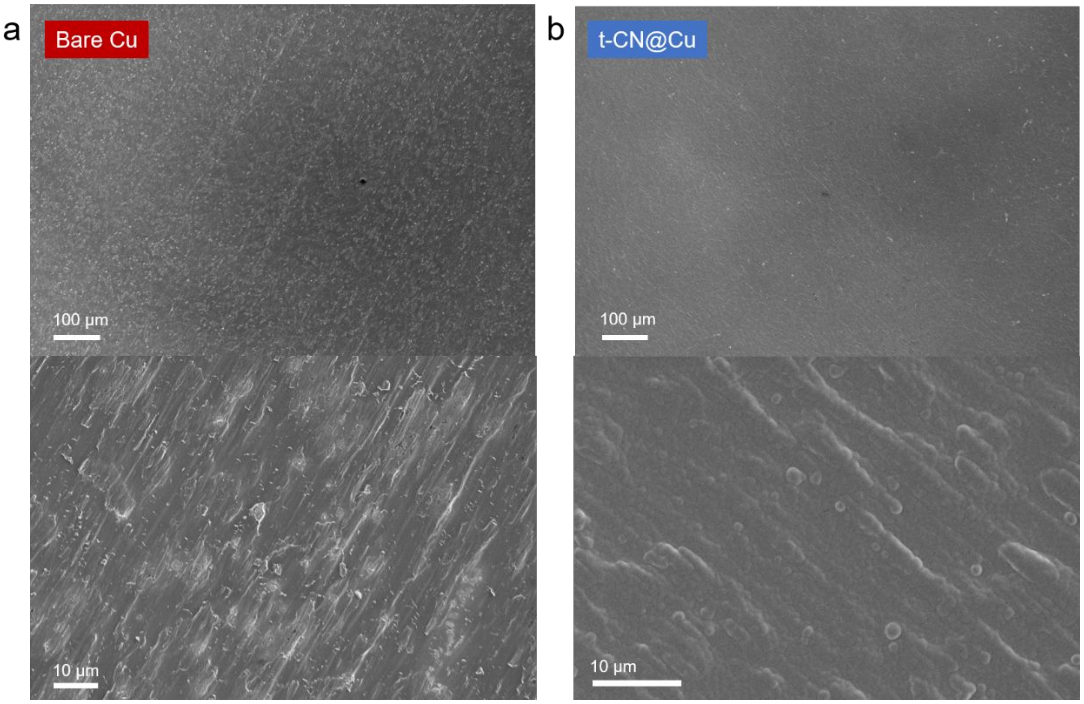

Figure S4 | SEM images of (a) bare Cu substrate and (b) t-CN@Cu sample.

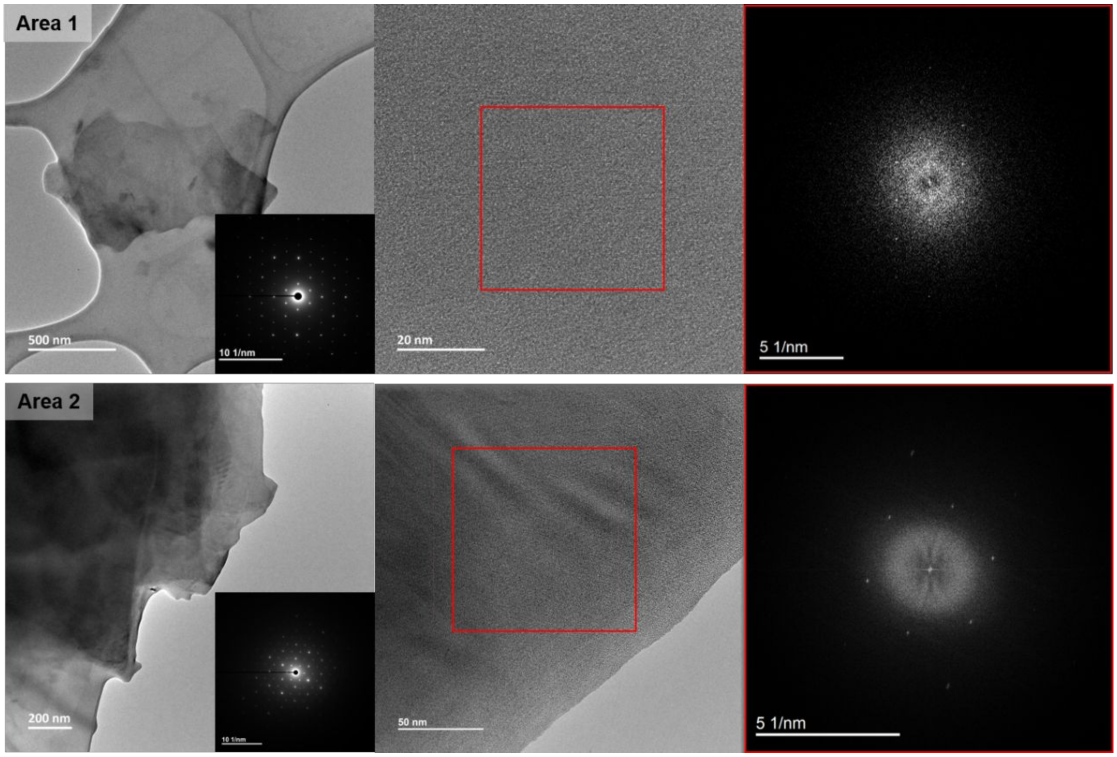

Figure S5 | HR-TEM images of different t-CN sheets with corresponding SAED and FFT images of the selected region.

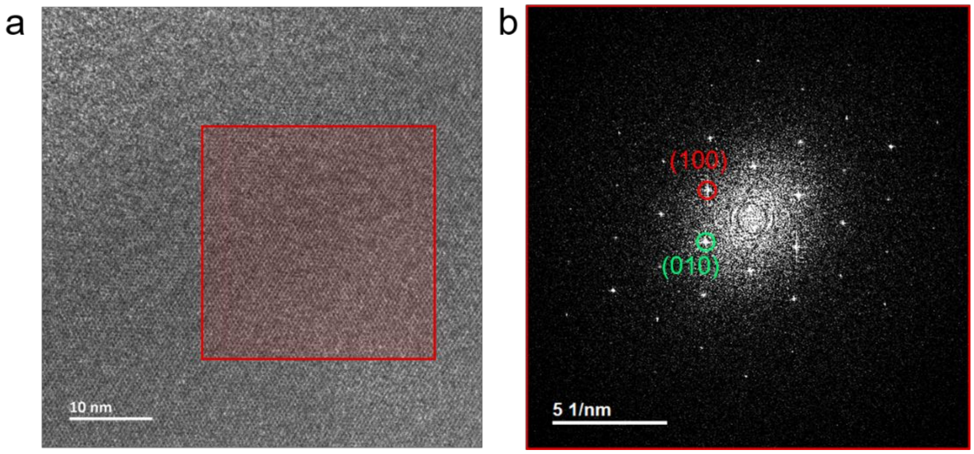

Figure S6 | (a) HR-TEM image of t-CN sheet with corresponding (b) FFT image of the selected region.

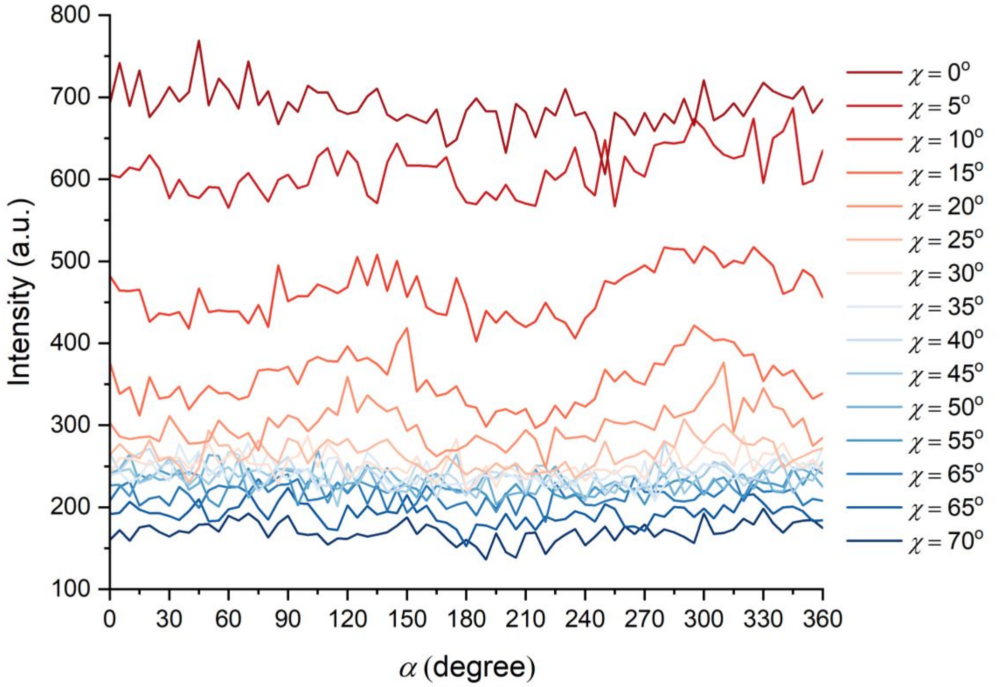

Figure S7 | Pole figure plots of (002) diffraction of t-CN@Cu sample.

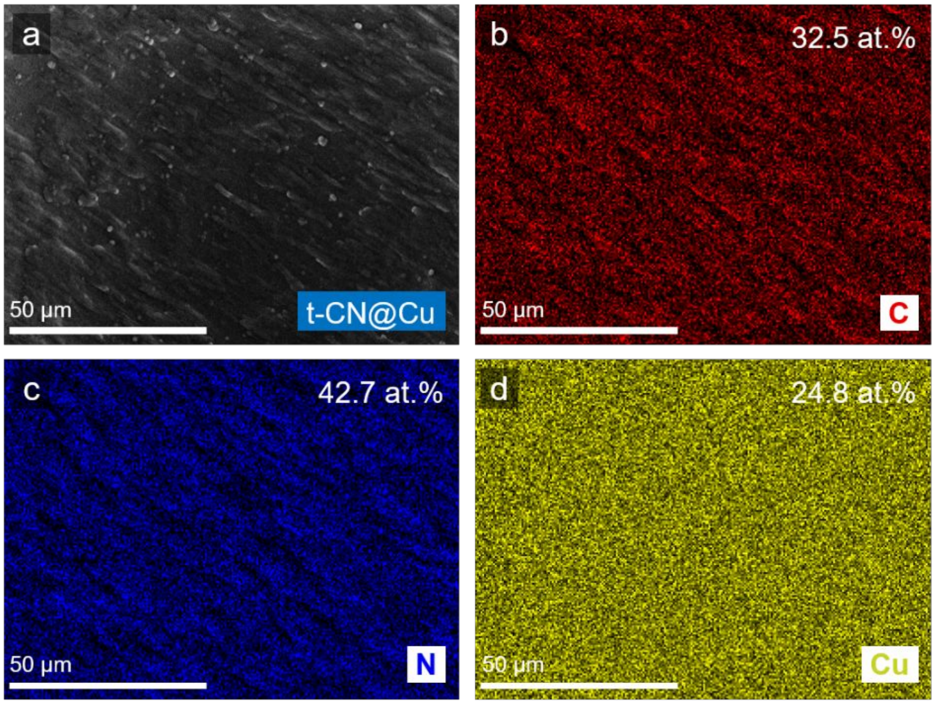

Figure S8 | SEM image and EDX mapping of t-CN@Cu sample. The C/N atomic ratio is 0.76 close to that of ideal triazine-based graphitic carbon nitride (C_3N_4).

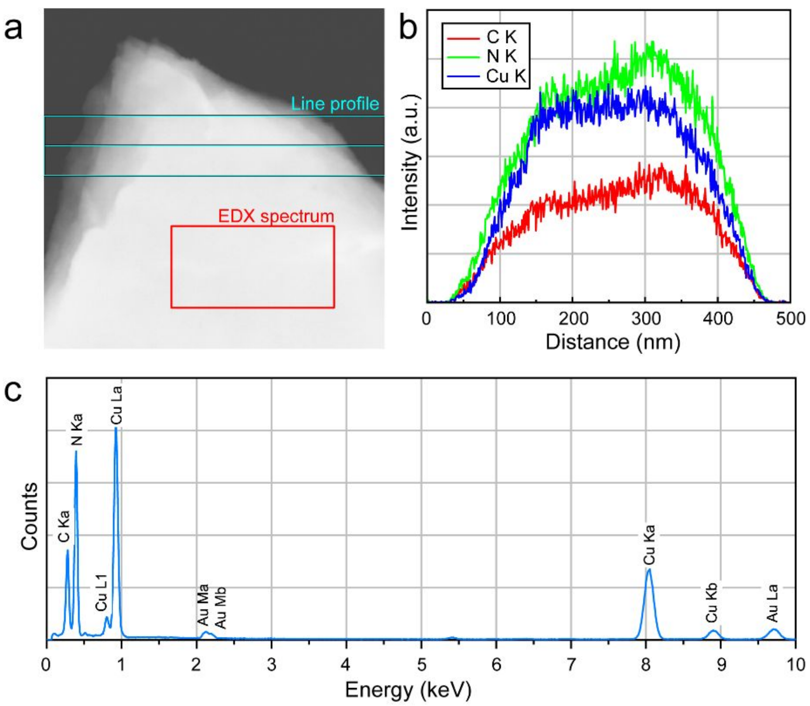

Figure S9 | The STEM image and corresponding spectra of selected regions. (a) the t-CN sheet of the collected EDX mappings in Figure 2a; (b) Line profile and (c) EDX spectra of the selected regions. The sample was loaded on a gold microgrid.

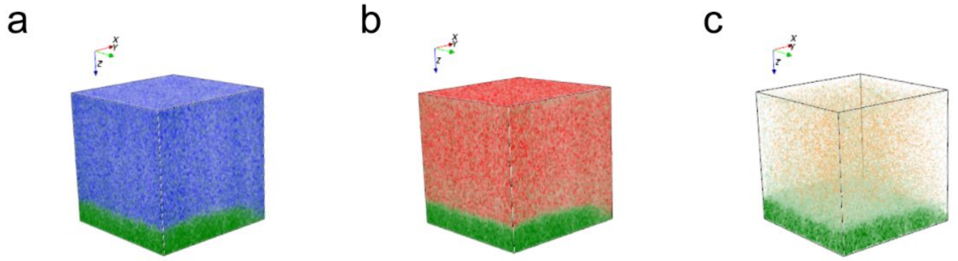

Figure S10 | Three-dimensional (3D) overlay figures of selected CuN_xC_y fragmentsfrom ToF-SIMS (green = Cu^-, blue = $CuNC^-$, red = $CuC_3N_3^-$ and yellow = $C_3N_3^-$). Overlay figures of (a) Cu^- and $CuNC^-$, (b) Cu^- and $CuC_3N_3^-$ and (c) Cu^- and $C_3N_3^-$.

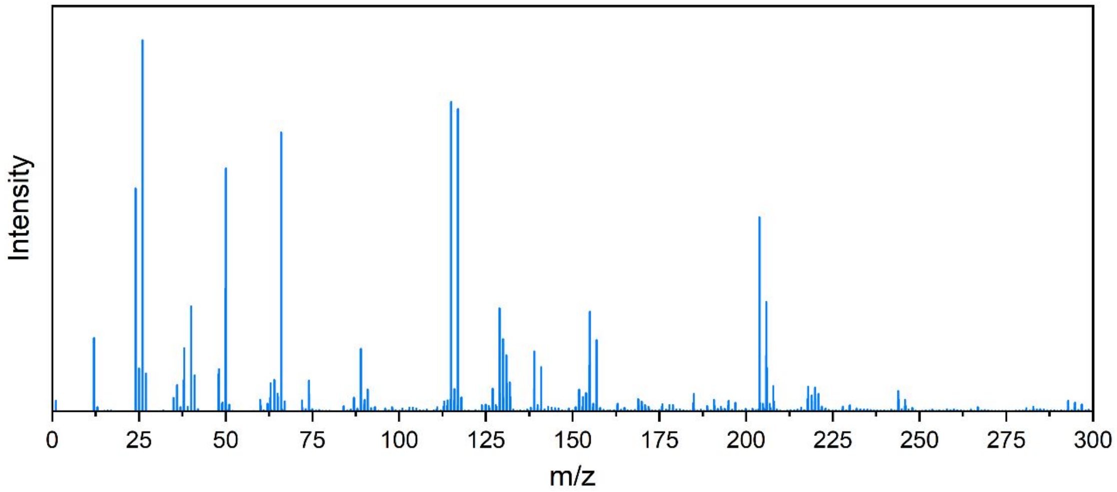

Figure S11 | ToF-SIMS spectrum of t-CN@Cu sample. The main signals are assigned to triazine-based units and $C_xN_yCu_z$ fragments, but the typical fragments of heptazine-based units are not pronounced. This result indicates the triazine-based structure and uniform dispersion of Cu atoms.[1]

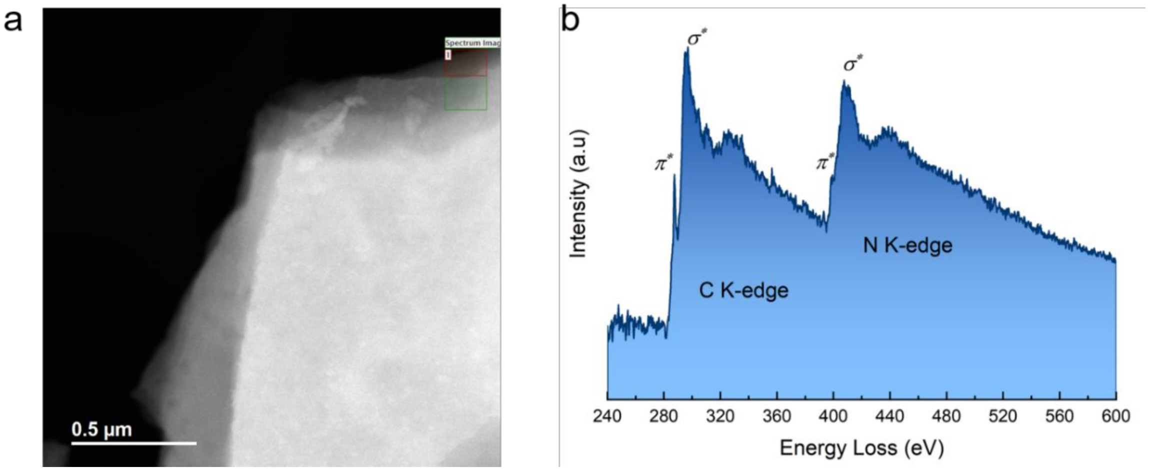

Figure S12 | (a) ADF-STEM image and corresponding (b) EELS spectrum of the t-CN layer. The C/N atomic ratio is ~0.77.

Table S1 | EELS analysis of the spectrum in Figure S9b.

Element	Shell	Signal (e^-)	Rel. comp. (/N)	X-section (barns)	X-section Model
C	K	1450.0 k ± 3.0 k	0.77	6.1 k ± 0.3 k	Hartree-Slater
N	K	1376.0 k ± 2.0 k	1.00	4.5 k ± 0.2 k	Hartree-Slater

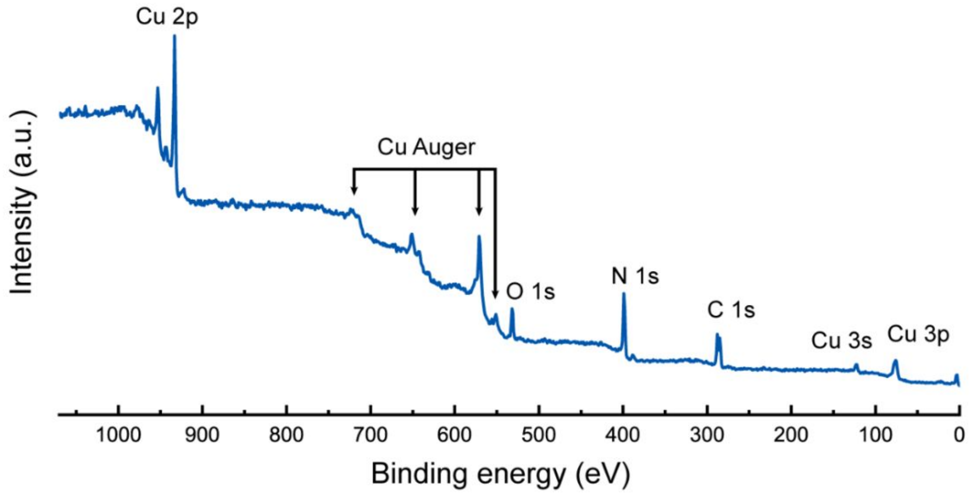

Figure S13 | The XPS spectrum of t-CN@Cu. The atomic C/N ratio is about 0.78, which is aligned with EDX results (Based on the C1s peak at 287.94). The atomic Cu/N ratio is about 0.36 as shown in **Table S1**.

Table S2 | Atomic ratio on surface (by XPS) for t-CN@Cu.

Elements	Orbitals	Positions (eV)	PP At. %	Rel. comp. (/N)
C	1s	287.94	36.33	0.78
N	1s	398.65	46.59	1.00
Cu	2p 3/2	932.69	17.08	0.36

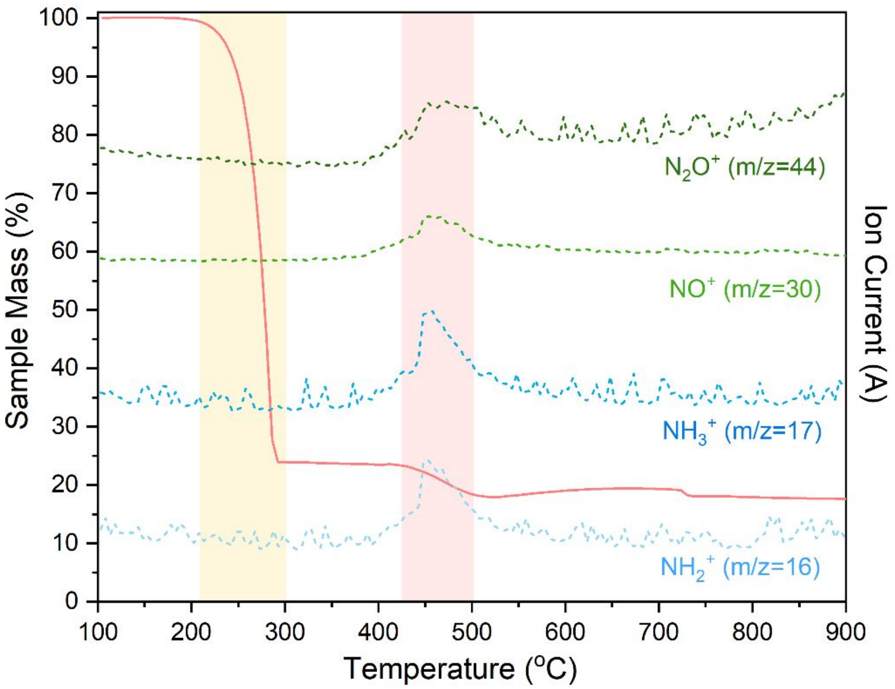

Figure S14 | TGA-MS results of melamine. The first mass loss region before 300 °C is attributed to the sublimation of melamine; the second mass loss between 420 °C and 500 °C is for the condensation of melamine. The ionic currents of NH_2^+ (m/z=16, light blue), NH_3^+ (m/z=17, dark blue), NO^+ (m/z=30, light green) and N_2O^+ (m/z=44, dark green) corresponding to the release of NH_3 and the reactions between NH_3 and Cu oxides on the surface of bare Cu.

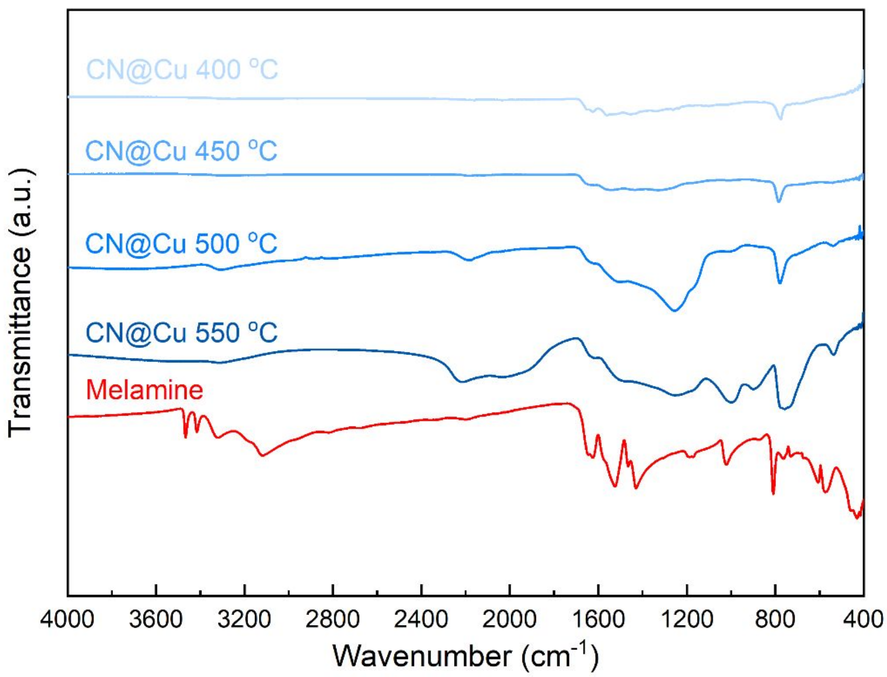

Figure S15 | The comparison of FT-IR spectra of precursor and samples prepared at different condensation temperatures.

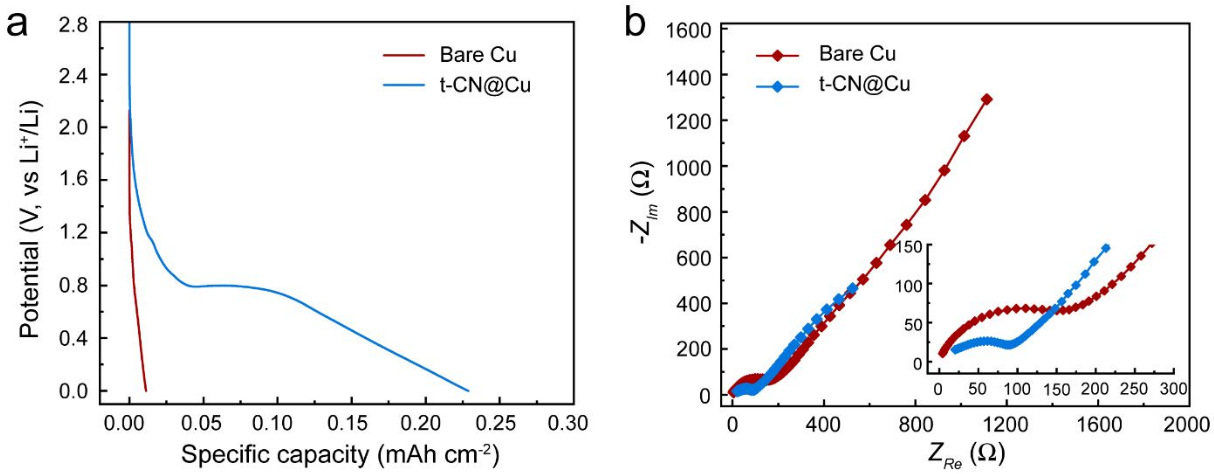

Figure S16 | The electrochemical activation of the t-CN@Cu electrode. (a) The initial lithiation of bare Cu and t-CN@Cu electrodes to 0 V (vs Li^+/Li) at a constant current density of 0.1 mA cm^{-2}; (b) EIS response of bare Cu and t-CN@Cu electrodes after activation cycles.

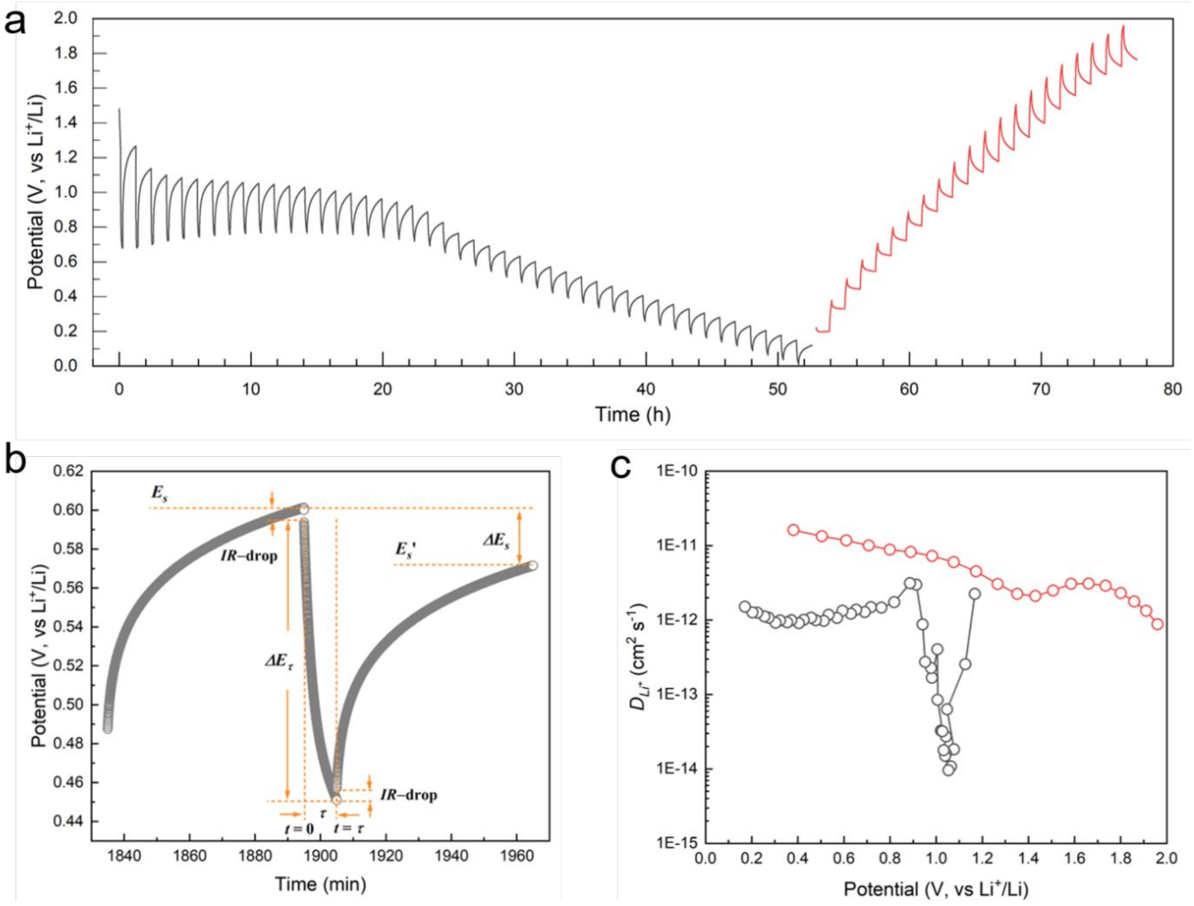

Figure S17 | GITT measurements of t-CN@Cu electrode. (a) GITT curves of t-CN@Cu electrode upon the initial lithitation cycle as a function of time in the potential range of 0.0-2.0 V (vs Li^+/Li); (b) A selected single titration of GITT measurement at around 0.55-0.60 V (vs Li^+/Li) upon lithiation process; (c) The chemical diffusion coefficients ($D_{Li}{}^+$, cm^2 s^{-1}) versus potential (V, vs Li^+/Li) of t-CN@Cu electrode in the initial lithitation cycle.

Note:

A single titration of the GITT measurement of t-CN@Cu electrode as a function of time is shown in Figure S7a with schematic labelling of different parameters. The values of chemical diffusion coefficients are determined based on the Fick's Second Law of Diffusion, and the approximately simplified equation for $D_{Li}{}^+$ is written as:

$$D_{Li^+} = \frac{4d^2}{\pi\tau}\left(\frac{\Delta E_S}{\Delta E_\tau}\right)^2$$

Particularly, d is the thickness of t-CN layers, which is approximated to the diffusion distance across a 2D film.

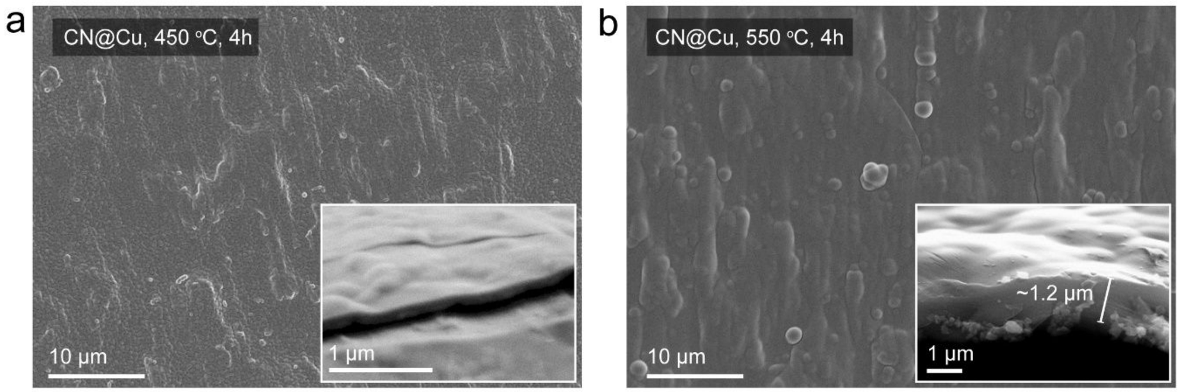

Figure S18 | SEM images of CN@Cu samples prepared at different temperatures. The surface and cross-section view (inset) of the samples prepared at (a) 450 °C and (b) 550 °C.

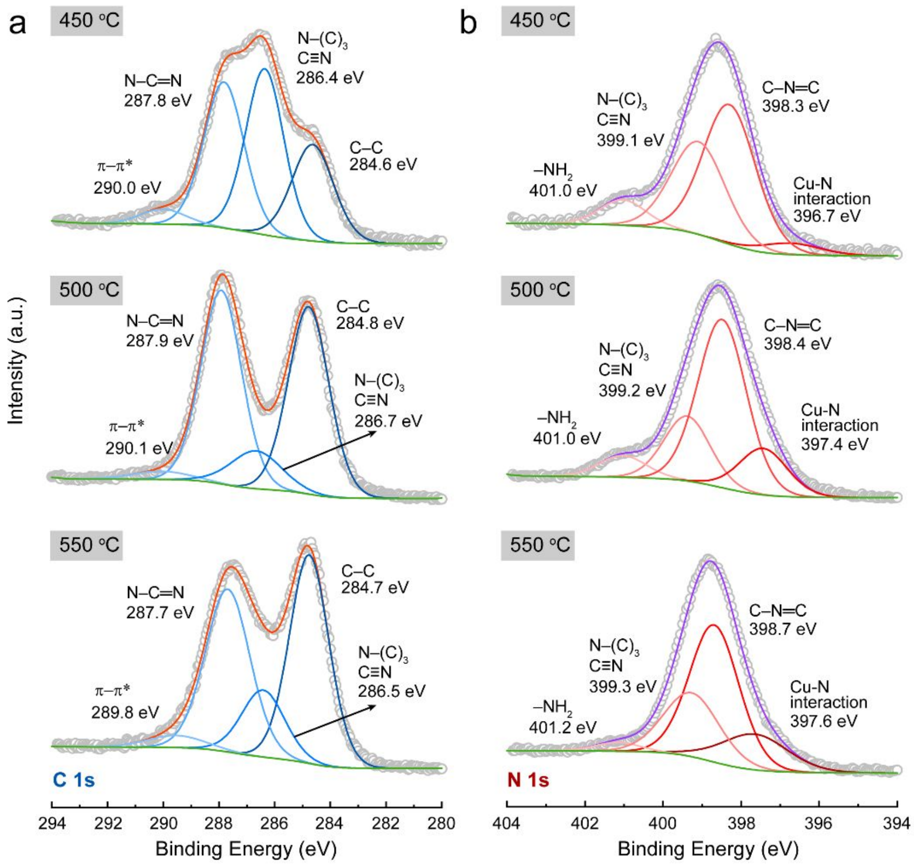

Figure S19 | XPS spectra of CN@Cu samples prepared at different temperatures. The XPS spectra of (a) C 1s and (b) N 1s. (top: 450 °C; middle: 500 °C; bottom: 550 °C)

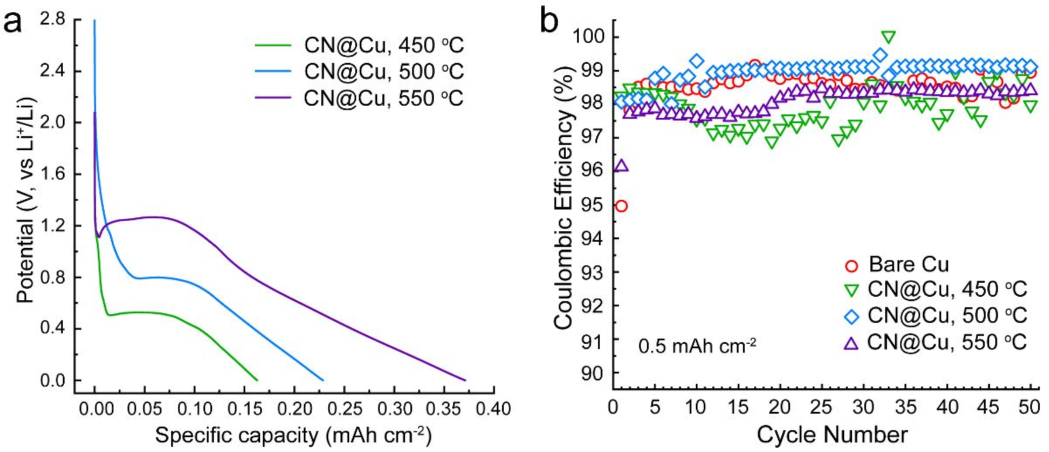

Figure S20 | The comparison of electrochemical Li storage behavior of CN@Cu electrodes prepared at different temperatures. (a) The activation curve and (b) cycling performance of different electrodes.

Note:

The differences between the CN@Cu samples prepared at different temperatures (450 ℃, 500 ℃ and 550 ℃) were investigated. As shown in the SEM images of the CN@Cu samples prepared at different temperatures (Figure S18), the CN layers at different temperatures are all evenly covered on the substrates, while the functional groups and degree of polymerization are varied. According to the FT-IR spectra in the supporting information (Figure S15), the CN prepared at 450 ℃ is low-polymerized as the weak vibrations at the range of 1100~1800 cm^{-2}, which are ascribed to the C−N of condensed triazine-based carbon nitride phase. As shown in the Figure S19, more contribution from C≡N terminal groups is observed in the XPS spectra of C1s and N1s of the sample prepared at 450 ℃, indicating the existence of ring-open structures and the low polymerization of CN. At a higher deposition temperature of 550 ℃, the change in the vibrations as shown in the FT-IR spectra reveals the depolymerization of t-CN phase (Figure S15). The XPS results show differences in the peak contributions between the sample prepared at 500 ℃ and 550 ℃, confirming the chemical environment changed at the high temperature. In addition, the thicknesses of these three samples are also different, which is confirmed by their varied capacities of initial lithiation. As shown in the Figure S20a, these three samples show different plateau potentials and specific areal capacities. Less condensation can be considered as less oxidization, which is translated into a low redox potential of the sample prepared at 450 ℃. The depolymerization of the sample prepared at 550 ℃ creates complicate chemical structures at more oxidized state, exhibiting a different electrochemical behavior from t-CN prepared at 500 ℃. As shown in the Figure S20b, the t-CN sample performs the best cycling stability among all these samples. Therefore, 500 ℃ was selected as the optimal condition for the synthesis of t-CN material in this study.

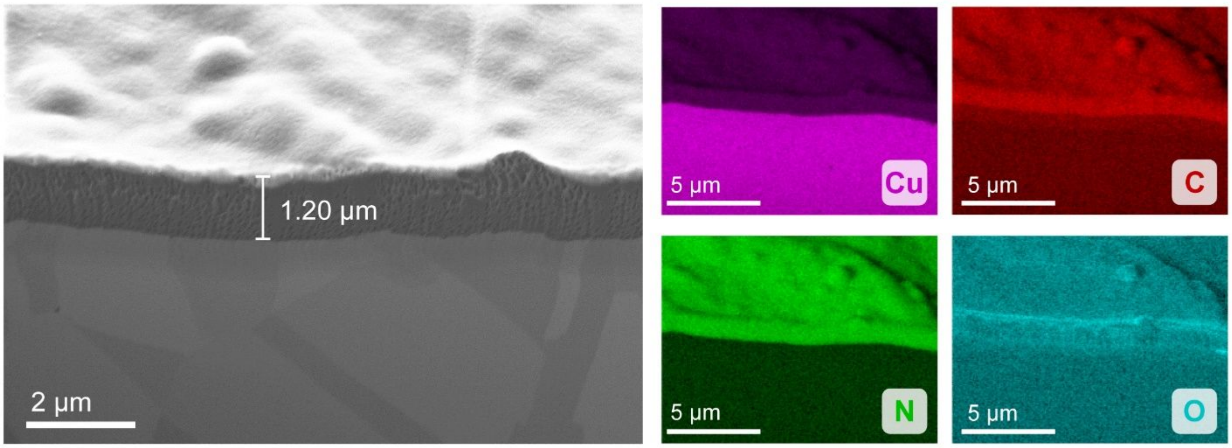

Figure S21 | The FIB-SEM images of the cross-section view of activated t-CN@Cu. The thickness of the t-CN interphase increases from 0.70 μm to 1.20 μm after activation. The corresponding elemental mapping images show that the electrolyte-derived SEI (containing O) mainly distributes on the interface between t-CN and electrolyte. This result indicates that the desolvation of Li^+ occurs out of the t-CN interphase and the continuous decomposition of electrolyte can be suppressed upon cycling.

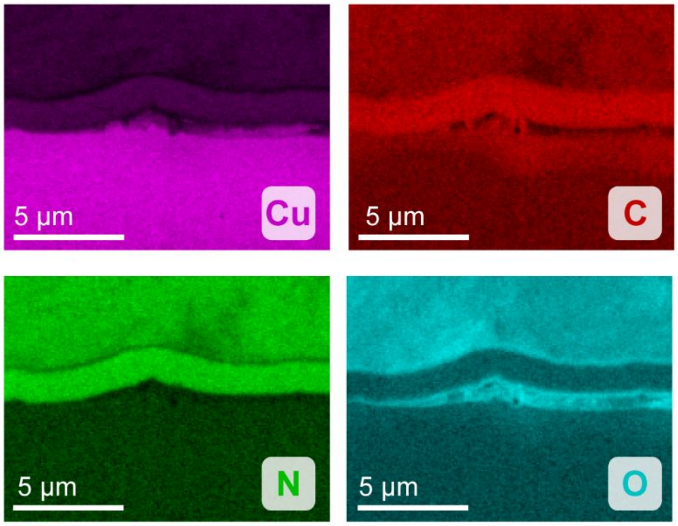

Figure S22 | EDX mapping of the cross-section view of t-CN@Cu electrode after 0.5 mAh cm^{-2} Li deposition (Figure 3d, inset)

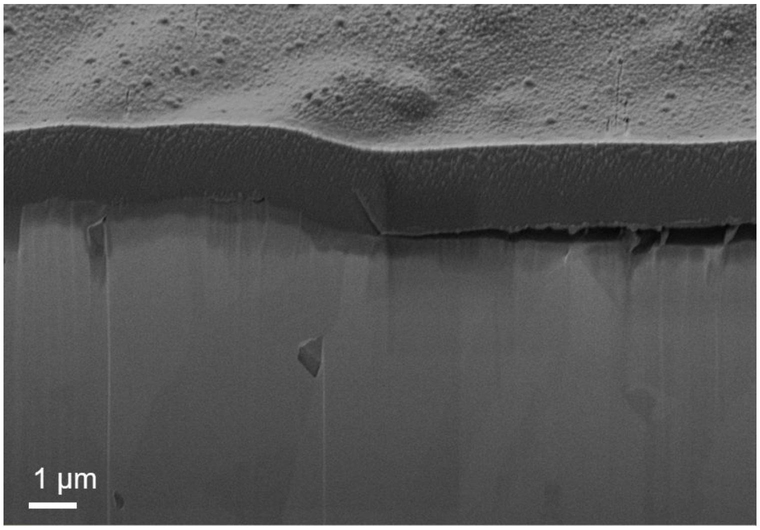

Figure S23 | FIB-SEM images of the cross-section view of t-CN@Cu electrodes after Li stripping. The thickness of the t-CN interphase is ~1.50 μm.

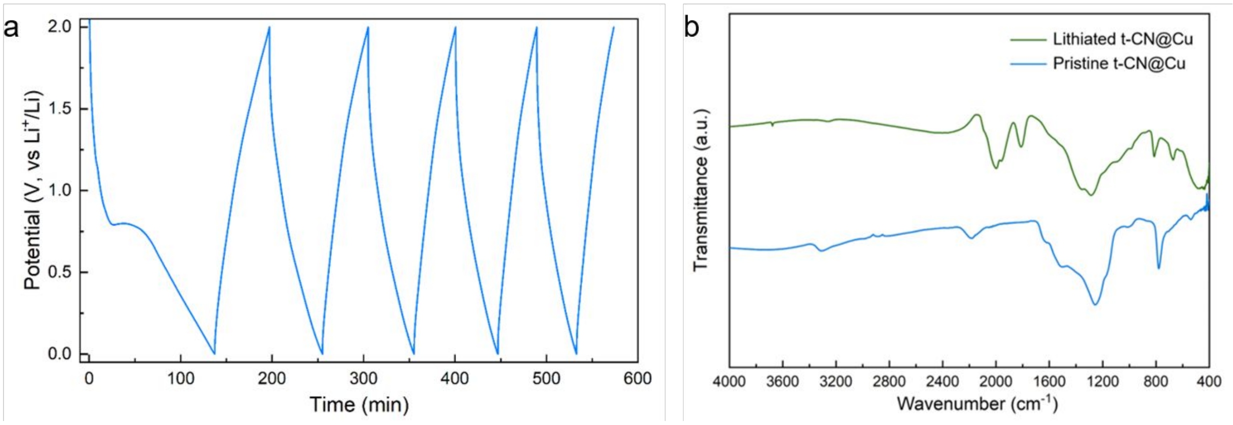

Figure S24 | The activation of t-CN@Cu electrode. (a) Galvanostatic charge/discharge curves of initial activation process; (b) FT-IR spectra of pristine and lithiated t-CN@Cu electrode.

Note:

The current density is 0.1 mA cm^{-2}. The t-CN@Cu electrode exhibits a specific capacity of 0.23 mAh cm^{-2} with a plateau at around 0.8 V (vs Li^+/Li), which is absent in the case of the bare Cu electrode. The formation of Li−N=C or Li−C=N species were observed in the activated electrode by FT-IR spectroscopy as a set of strong vibration bands centred near 2000 cm^{-1}, which are similar to the spectral features in this region of lithium nitrile.[2] We cannot exclude that the active N sites react with Li^+ and electrons, contributing partially to the irreversible capacity in the plateau region, which occurs in the first charging process.

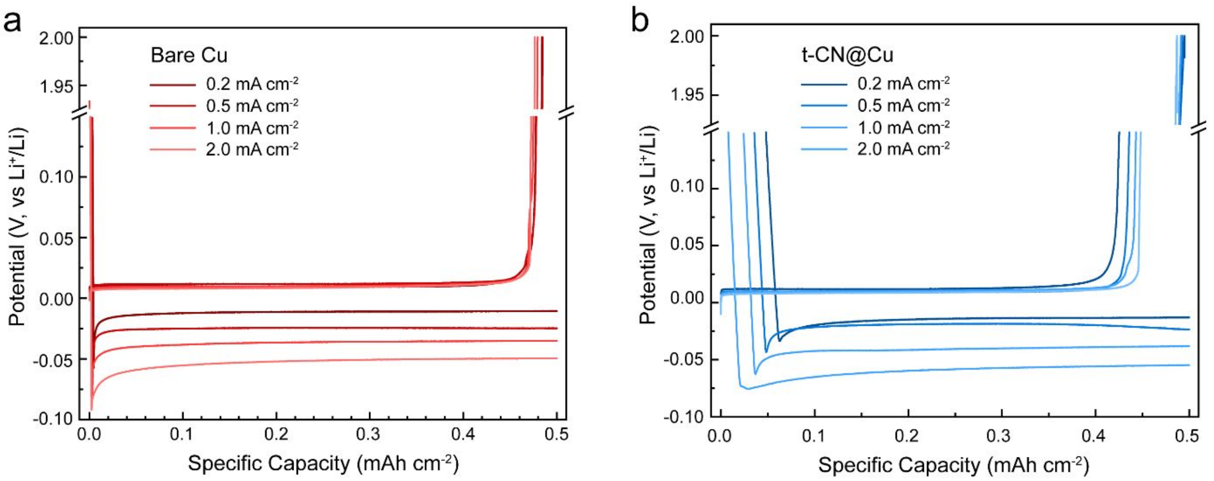

Figure S25 | Galvanostatic charge/discharge curves of (a) bare Cu and (b) t-CN@Cu electrodes at various discharge current densities.

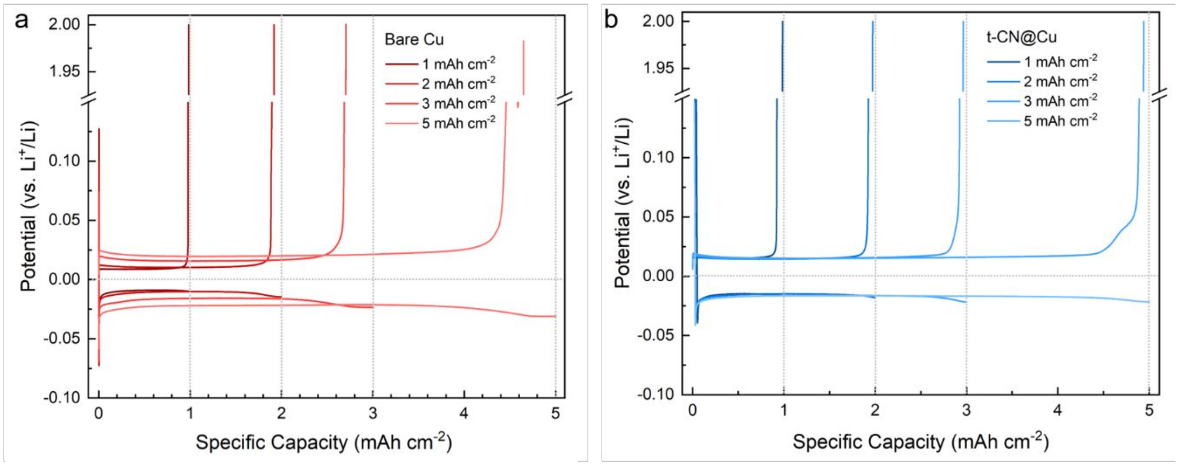

Figure S26 | Galvanostatic charge/discharge curves of (a) bare Cu and (b) t-CN@Cu electrodes with various specific areal capacities at the current density of 0.5 mA cm^{-2}.

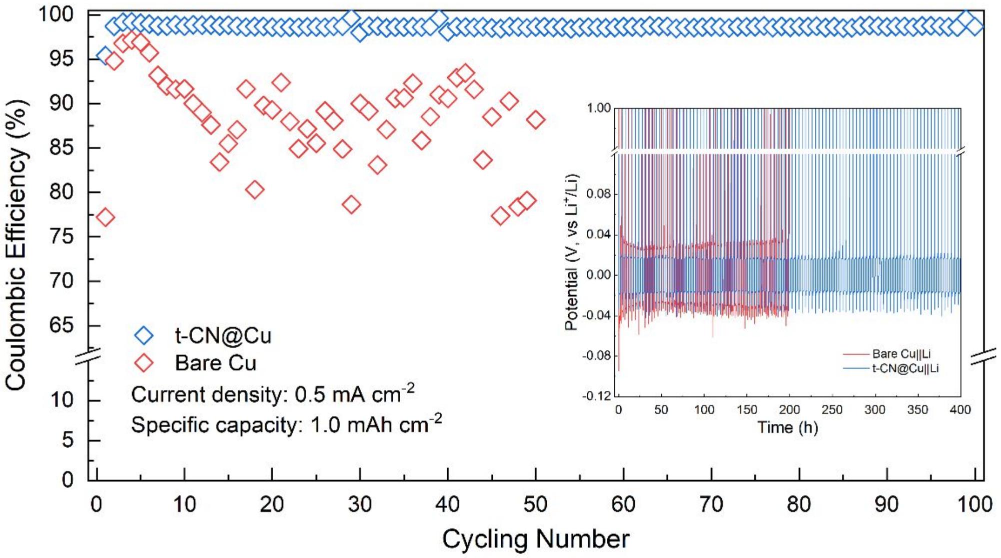

Figure S27 | The cycling performance of bare Cu||Li and t-CN@Cu||Li cells after the multi-capacity cycling tests. Inset: potential vs time plot of corresponding charge/discharge cycling.

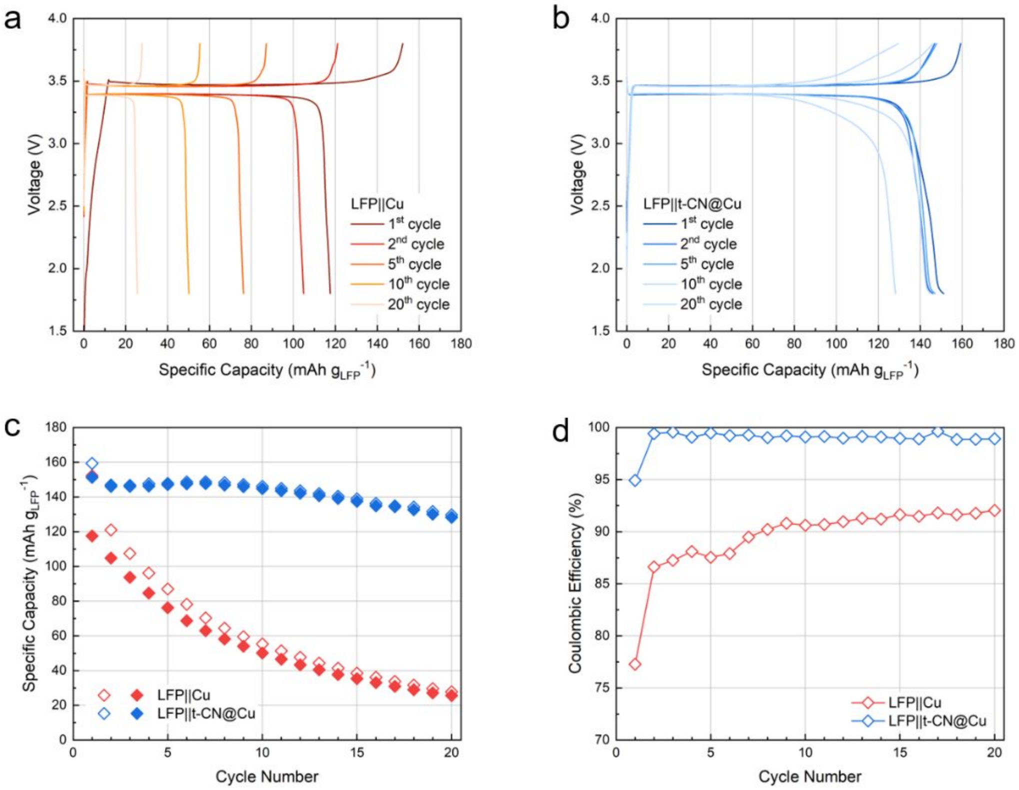

Figure S28 | Full cell performance of LFP||Cu and LFP||t-CN@Cu batteries. Galvanostatic charge/discharge curves of (a) LFP||Cu and (b) LFP||t-CN@Cu at 0.5C (1C = 170 mA g_{LFP}^{-1}); (c) The cycling performance of LFP||Cu and LFP||t-CN@Cu batteries; (d) The Coulombic efficiencies of LFP||Cu and LFP||t-CN@Cu batteries during cycling.

Reference

(1) Hou, Y.; Fang, Y.; Zhou, Z.; Hong, Q.; Li, W.; Yang, H.; Wu, K.; Xu, Y.; Cao, X.; Han, D.; et al. Growth of Robust Carbon Nitride Films by Double Crystallization with Exceptionally Boosted Electrochemiluminescence for Visual DNA Detection. *Adv. Optical Mater.* **2023**, *11* (6), 2202737. DOI: https://doi.org/10.1002/adom.202202737.

(2) Veith, G. M.; Baggetto, L.; Adamczyk, L. A.; Guo, B.; Brown, S. S.; Sun, X.-G.; Albert, A. A.; Humble, J. R.; Barnes, C. E.; Bojdys, M. J.; et al. Electrochemical and Solid-State Lithiation of Graphitic C_3N_4. *Chem. Mater.* **2013**, *25* (3), 503-508. DOI: 10.1021/cm303870x.
